# Detection of antimicrobial resistance-associated proteins by titanium dioxide-facilitated intact bacteria mass spectrometry†
†Electronic supplementary information (ESI) available. See DOI: 10.1039/c7sc04089j


**DOI:** 10.1039/c7sc04089j

**Published:** 2018-01-18

**Authors:** Yingdi Zhu, Natalia Gasilova, Milica Jović, Liang Qiao, Baohong Liu, Lysiane Tissières Lovey, Horst Pick, Hubert H. Girault

**Affiliations:** a Laboratoire d’Electrochimie Physique et Analytique , École Polytechnique Fédérale de Lausanne , Rue de l’industrie 17 , CH-1951 Sion , Switzerland . Email: hubert.girault@epfl.ch; b ISIC-GE-VS , École Polytechnique Fédérale de Lausanne , Rue de l’industrie 17 , CH-1951 Sion , Switzerland; c Department of Chemistry , Fudan University , Handan Road 220 , 200433 Shanghai , China; d ICH , Hôpital du Valais, Avenue du Grand Champsec 86 , CH-1951 Sion , Switzerland; e Laboratoire de Chimie Biophysique des Macromolécules , École Polytechnique Fédérale de Lausanne , CH-1015 Lausanne , Switzerland

## Abstract

Titanium dioxide-modified target plates were developed to enhance intact bacteria analysis by matrix-assisted laser desorption/ionization time-of-flight mass spectrometry. The plates were designed to photocatalytically destroy the bacterial envelope structure and improve the ionization efficiency of intracellular components, thereby promoting the measurable mass range and the achievable detection sensitivity. Accordingly, a method for rapid detection of antimicrobial resistance-associated proteins, conferring bacterial resistance against antimicrobial drugs, was established by mass spectrometric fingerprinting of intact bacteria without the need for any sample pre-treatment. With this method, the variations in resistance proteins’ expression levels within bacteria were quickly measured from the relative peak intensities. This approach of resistance protein detection directly from intact bacteria by mass spectrometry is useful for fast discrimination of antimicrobial-resistant bacteria from their non-resistant counterparts whilst performing species identification. Also, it could be used as a rapid and convenient way for initial determination of the underlying resistance mechanisms.

## Abstract 2

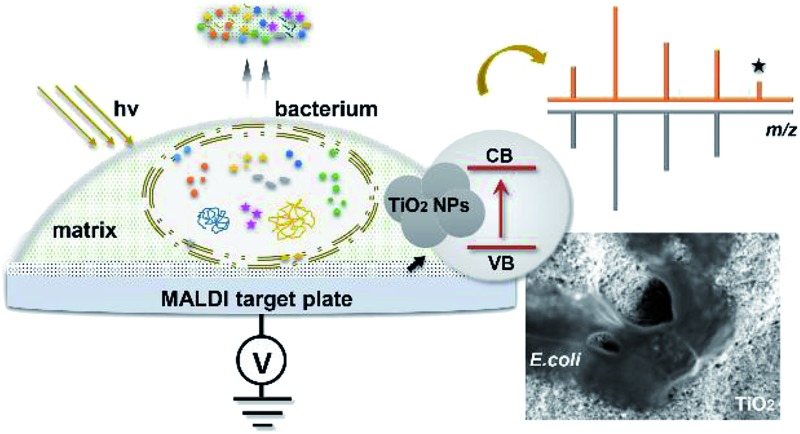
TiO_2_-facilitated MALDI–TOF-MS was proposed to improve intact bacteria fingerprinting, allowing rapid and convenient antimicrobial resistance-associated protein detection during bacteria identification.

## Introduction

Infectious diseases caused by pathogenic bacteria are serious threats to human health. Misuse and overuse of antimicrobial drugs over many years have led to the emergence of antimicrobial resistance among microbes worldwide.^1^ For fast diagnosis and efficient treatment, it is crucial to perform pathogen identification and a rapid analysis of their antimicrobial resistance phenotypes. With the ability to generate characteristic mass spectral fingerprints directly from intact bacteria, matrix-assisted laser desorption/ionization time-of-flight mass spectrometry (MALDI–TOF MS) provides a rapid method for bacteria identification (*e.g.* ∼30 min for 48 samples) and has received clearance from the US Food and Drug Administration (FDA).^2^,^3^ Commercial systems, including Vitek MS (bioMérieux) and MALDI Biotyper (Bruker Daltonics), have been installed and constantly used in many hospitals. Meanwhile, antimicrobial resistance detection in hospitals still mainly relies on antimicrobial drug culture approaches like the broth (or agar) microdilution method and disk diffusion method, which need several hours or even several days.^4^ Therefore, performing a complete clinical diagnosis remains a lengthy process.

In addition to the classical culture-based methods, several new strategies have been proposed for antimicrobial resistance detection. Examples include nucleic acid-based resistance gene detection,^5^ single-cell morphological analysis,^6^ surface-enhanced Raman spectroscopic biomarker detection,^7^ atomic force microscope cantilever-based nanomechanical sensors,^8^*etc.*

Recently, continuous efforts have been made to explore the potential of MALDI–TOF MS for rapid antimicrobial resistance analysis. Related studies were mainly carried out with three approaches. The first one is an indirect evaluation by detection of resistance enzyme activity, such as the degradation of β-lactam antibiotics through hydrolysis (mass increased by 18 Da) by β-lactamases^9^ and the alternation of rRNA through methylation (mass increased by 14 Da) by rRNA methyltransferase.^10^ This is a fast method for resistance mechanism investigation, but is limited to certain enzyme-related resistance types. The second approach is an isotope labelling-bacteria culture method, with the appearance of peak mass shifts in bacterial fingerprinting patterns if a resistant strain is incubated with culture medium supplemented with stable (non-radioactive) isotope labelled amino acids and corresponding antibiotics.^11^,^12^ Based on the machinery of protein biosynthesis, this approach is applicable to determining bacterial resistance or susceptibility to a wide range of antibiotics, but limited by the need for special isotope labelled culture medium. The third one is also a culture-based method, in which semi-quantitative MALDI–TOF MS using an internal standard is employed to investigate bacterial growth status with the presence or absence of antibiotics by measuring the quantity of biomass within a spectrum.^13^,^14^ This method has been demonstrated to be feasible for different antibiotic classes/bacterial species combinations. In addition to the above three approaches, bacteria subtyping assays have also been conducted to study the correlation between antibiotic-susceptible and resistant strains by comparing their fingerprint patterns. For example, it has been used to discriminate major methicillin-resistant *Staphylococcus aureus* lineages^15^ and to identify vancomycin-resistant *Enterococcus* spp.^16^

Proteins encoded by antimicrobial resistance genes are directly involved in bacterial resistance process against antimicrobial drugs.^17^ Antimicrobial resistance can be analysed by tracing these resistance-associated proteins within bacterial cells. Ideally, they should be read out directly from MALDI–TOF MS fingerprint patterns of intact bacteria without any sample pre-treatment, a useful procedure that would be comparable to fast bacteria identification. But many of those proteins are large ones (>15 000 Da) expressed in low abundance, and are difficult to detect directly from intact cells by classic MALDI–TOF MS measurements, which typically focus on smaller proteins (<15 000 Da) expressed in high abundance.^18^ Until now, to the best of our knowledge no studies have reported the success of this procedure, as pointed out in a review by Walkova *et al.*^19^ In order to detect these resistance-associated proteins, preparatory extraction and enrichment processes are required prior to their identification by MS, which is labour-intensive and time-consuming.^20^–^22^ Very recently, a surrogate marker around 11 kDa was detected from carbapenem-resistant bacteria strains containing *bla*_*KPC*_-harboring plasmids by a MALDI–TOF MS fingerprinting approach. But it is a particularly small protein and an additional step of protein extraction was required prior to MS analysis.^23^

Herein, we have developed a MALDI–TOF MS fingerprinting approach for intact bacteria analysis using photo-reactive titanium dioxide (TiO_2_)-modified target plates, providing access to a high mass range with enhanced detection sensitivity. TiO_2_-modified target plates or more generally metal oxide-modified plates have been used for many different MALDI–TOF MS applications.^24^,^25^ In the current work, the rationale of the proposed approach is to take advantage of the photo-reactivity of TiO_2_ to destroy bacterial cell membranes and to facilitate inner component desorption/ionization. Such experimental improvement makes feasible a direct fast read out of resistance-associated proteins from intact bacteria cells without any sample pre-treatment.

## Results and discussion

### TiO_2_-facilitated intact bacteria MALDI–TOF MS fingerprinting

One important factor affecting MALDI–TOF MS measurements is the choice of matrix. Sinapinic acid was utilized as a matrix throughout this work, as it provides satisfying reproducibility and facilitates the detection of large proteins (Part S1, ESI†). Based on our experience in designing photo-reactive TiO_2_-modified target plates for inducing in-source electrochemical reactions,^26^,^27^ we have developed here a plate able not only to absorb bacteria on a porous structure but also to lyse them by photocatalytic oxidation, improving intact bacteria fingerprinting in a broad mass range as demonstrated below.

**Fig. 1:**
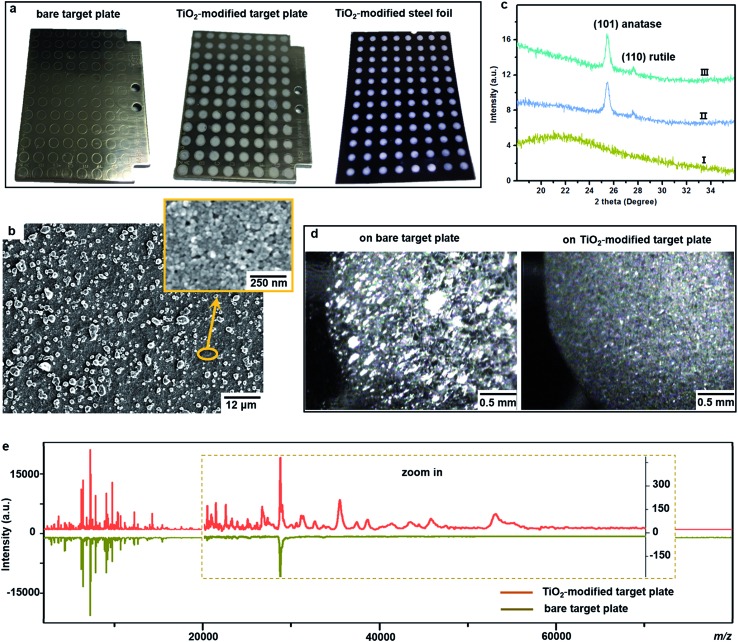
(a) Photos of a classic bare stainless steel target plate (MSP 96 ground steel MALDI target, Bruker Daltonics), a TiO_2_ NP-modified target plate and a piece of TiO_2_ NP-modified stainless steel foil, which was affixed onto a bare target plate before MALDI–TOF MS measurements; (b) scanning electron microscope image of a TiO_2_ NP layer after sintering at 400 °C; (c) X-ray powder diffraction patterns of (I) the steel substrate, (II) TiO_2_ NPs on the steel surface before sintering at 400 °C and (III) TiO_2_ NPs on the steel surface after sintering at 400 °C; (d) microscope image of intact bacteria (*E. coli*)/sinapinic acid matrix crystals (the shining clusters) on a spot of a classic bare target plate and a TiO_2_ NP-modified target plate; (e) MALDI–TOF MS fingerprint patterns (each was averaged from three replicates) of intact *E. coli* in a mass range of *m*/*z* = 2000–80 000 obtained using a classic bare and a TiO_2_ NP-modified target plate.

This target plate was prepared by depositing an aqueous suspension of TiO_2_ nanoparticles (NPs) on the spots (3 mm diameter) of a classic bare stainless steel target plate, or by dropping TiO_2_ suspension as an array of spots on a stainless steel foil (20 μm thick), which was afterwards affixed onto a bare target plate by an adhesive tape (Fig. 1a). The TiO_2_ NPs were subsequently thermally or photonically sintered. The sintered NPs exhibited strong adherence to the steel substrate and provided a stable support layer (∼3 μm thick) for the bacteria and matrix, with small particles (of 20–25 nm size) densely covering the bottom and large particles (of 0.5–3 μm size) observed on the surface (Fig. 1b). The TiO_2_ used is a commercial P25 nanopowder, a mixture of anatase (80%) and rutile (20%) crystalline phases. The anatase is more photo-reactive than the rutile, but the latter is more thermodynamically stable. The crystalline phases of TiO_2_ were not changed after sintering, and the corresponding X-ray powder diffraction patterns are shown in Fig. 1c. Compared to bare steel target spots, the spots with TiO_2_ NPs had rough and mesoporous surfaces (see the surface roughness profiles in Part S2, ESI†), with a larger surface area and lower water contact angle (decreased from 70° to 38°, Part S3, ESI†). As the surface of the TiO_2_ spots are more hydrophilic than the steel substrate, this kind of TiO_2_-modified plate can be used as “AnchorChip” targets. Upon deposition, bacteria cells (mostly of 0.2–2 μm size) entered into the porous TiO_2_ NPs structure. Due to the high affinity between the bacterial membrane and TiO_2_,^28^ the cells tended to be absorbed on the surface of TiO_2_. Matrix drop casting consequently led to the formation of fine and well dispersed bacteria/matrix crystals, highly favourable for an efficient desorption/ionization process (Fig. 1d). MALDI–TOF MS analysis of intact *Escherichia coli* (*E. coli*, strain DH5α) yielded much higher quality fingerprint patterns using a TiO_2_-modified target plate in comparison with a bare steel target plate (Fig. 1e). Such significant improvement, especially in the mass range *m*/*z* = 15 000–60 000, could not be solely caused by the high quality of bacteria/matrix co-crystals resulting from the mesoporous spots’ surface. It could also be explained by the ability of TiO_2_ to destroy the bacterial cell membrane and to improve analyte desorption/ionization due to its well-known photo-reactivity.^28^,^29^

**Fig. 2:**
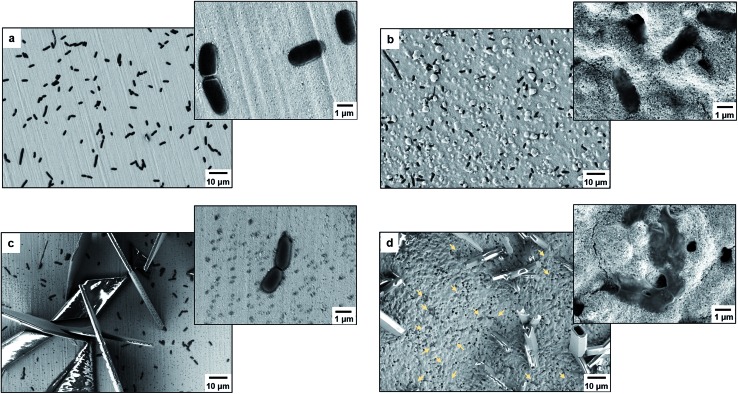
Scanning electron microscope images of *E. coli*: (a) deposited on a spot of the bare target plate with no matrix covering and no MALDI laser irradiation (no TiO_2_/no matrix/no laser irradiation), (b) deposited on a spot of the TiO_2_ NP-modified target plate with no matrix covering and no MALDI laser irradiation (with TiO_2_/no matrix/no laser irradiation), (c) deposited on a spot of the bare target plate with matrix covering and with MALDI–TOF MS measurement (no TiO_2_/with matrix/with laser irradiation), (d) deposited on a spot of the TiO_2_ NP-modified target plate with matrix covering and with MALDI–TOF MS measurement (with TiO_2_/with matrix/with laser irradiation); the yellow arrows point to some of the *E. coli* cells. The matrix used is 15 μg mL^–1^ of sinapinic acid in 50/49.9/0.1 (v/v/v) acetonitrile/water/trifluoroacetic acid.

As proof of cell membrane disruption, the morphological changes of *E. coli* were visualized by scanning electron microscopy (Fig. 2). *E. coli* cells showed a straight, rod-like shape when they were deposited on the spots of a bare and a TiO_2_-modified target plate with no matrix covering and no MALDI laser irradiation (Fig. 2a and b). The spots with *E. coli* were then covered with matrix and underwent the running of a typical MS measurement (500 nitrogen laser shots on each sample spot, 20 Hz laser frequency). On the spots of the bare target plate, most *E. coli* cells (>95% according to microscopic observation) generally maintained their rod-like shape (Fig. 2c). The diameter of the laser beam used in the MALDI–TOF MS instrument (Bruker Microflex) is about 100 μm, 30 times smaller than the sample spot size (3 mm diameter). Thus, a typical MS measurement is accomplished with many “blind shots”, and only the cells exactly shot by the laser could be lysed. However, the situation was different on the spots with TiO_2_: most cells were seriously damaged with apparent deformation and membrane rupture, and the “melted” cells were embedded into the mesoporous spot surface (Fig. 2d). Interestingly, it was found that the crystal shape of the matrix on the spots with TiO_2_ was quite different from that on the bare spots (Fig. 2c and d). These microscopic observations show that the presence of TiO_2_ can indeed cause the disruption of more bacteria cells during MALDI–TOF MS measurements. With a band gap of 3.0–3.2 eV,^30^ TiO_2_ has strong light absorption in the UV range (Part S4, ESI†). Accordingly, during MS measurements, TiO_2_ absorbed energy from the nitrogen laser source (337.1 nm), generating electron–hole pairs, and triggering electron-transfer and radical reactions (see equations in Part S5, ESI†). The generated reactive oxygen species, like positive hole h^+^, hydroxyl radical ˙OH and peroxide H_2_O_2_, on the TiO_2_ surface caused oxidative disruption of the bacterial envelope, as previously reported.^31^,^32^ The disruption of more bacteria cells facilitated the detection of barely accessible inner cell components. The importance of oxidative cell disruption for enhancing bacteria MS analysis was confirmed by sample treatment with scavengers of these reactive oxygen species. Bacteria aqueous solutions containing different scavengers, *i.e.*, sodium oxalate, isopropanol and ferrocenemethanol, were deposited onto TiO_2_-modified target plates for MALDI–TOF MS measurements. The concentration of each scavenger was set to an appropriate value to eliminate its possible influence on bacterial cells (initial pH 7.44): 2 mM sodium oxalate (pH 7.87), 2 mM isopropanol (pH 7.11) and 0.4 mM ferrocenemethanol (pH 7.90).^32^,^33^ The MALDI–TOF MS analysis in the presence of these scavengers showed low quality bacterial fingerprint patterns throughout the mass range *m*/*z* = 2000–80 000 (Part S6, ESI†). In addition to disrupting bacteria cells, the high photo-reactivity of TiO_2_ favours efficient energy absorption from the laser source and transfer of this energy to matrix/analyte.^34^ This process occurs in addition to laser energy absorption directly by the matrix, and thus can facilitate analyte desorption/ionization even further. This was demonstrated by the analysis of bacterial protein extracts and standard protein mixtures. For the bacterial protein extracts, prepared according to the often-used ethanol/formic acid/acetonitrile extraction protocol, much higher quality MS patterns were observed using a TiO_2_-modified target plate than when using a classic bare one (Part S7, ESI†). For the standard protein mixtures, containing cytochrome c (∼12 kDa), myoglobin (∼17 kDa), bovine serum albumin (BSA, ∼66 kDa) and lactoferrin (∼82 kDa), the MS peak intensity of each protein was increased by the presence of TiO_2_. Consequently, the detection sensitivity was also improved, especially for the two hardly-ionized large proteins BSA and lactoferrin (Part S8, ESI†).

For comparison, we have tested the performance of non-photo-reactive nanomaterials like Al_2_O_3_ NPs (<50 nm in particle size) and SiO_2_ NPs (200 nm in particle size). They were shown to have detrimental effects on MS results for both standard protein mixtures and intact bacteria (Part S9 and S10, ESI†).

**Fig. 3:**
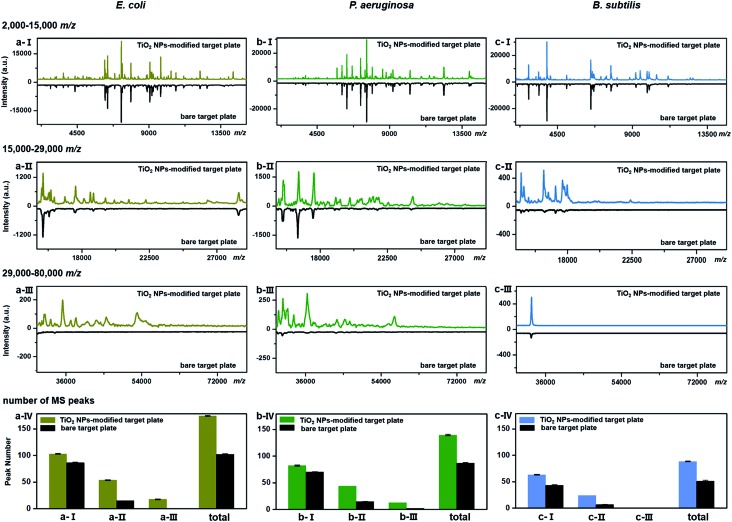
Comparison of a classic bare and a TiO_2_ NP-modified target plate for intact bacteria MALDI–TOF MS fingerprinting. The fingerprint patterns (each was averaged from three replicates) were generated from intact (a) *E. coli*, (b) *P. aeruginosa* and (c) *B. subtilis* in the mass range of (a/b/c-I) *m*/*z* = 2000–15 000, (a/b/c-II) *m*/*z* = 15 000–29 000 and (a/b/c-III) *m*/*z* = 29 000–80 000, with peak numbers counted in a/b/c-IV. During the peak number counting process, the threshold value of signal-to-noise ratio (S/N) of counted peaks was set as 3, and the threshold value of relative peak intensity was set as 2.0% for the mass range *m*/*z* = 2000–15 000 and 0.1% for the mass range *m*/*z* = 15 000–80 000. The number of bacterial cells on each sample spot was around 5 × 10^5^. All measurements were conducted under the exact same instrumental parameters.

TiO_2_-facilitated intact bacteria MALDI–TOF MS fingerprinting was further tested on different bacteria species. In addition to *E. coli* (strain DH5α), two more species, *i.e.*, *Pseudomonas aeruginosa* (*P. aeruginosa*, strain ATCC 27853) and *Bacillus subtilis* (*B. subtilis*, strain 168), were chosen as model analytes. All bacteria were measured in their intact whole state, without any preparatory protein extraction. Corresponding fingerprint patterns generated with a classic bare target plate and a TiO_2_-modified one with exactly the same measurement parameters are compared in Fig. 3a–c. Notably, each MALDI–TOF MS test in the present work was repeated three times. In each replicate, a freshly cultured bacteria strain was measured. Collected fingerprint patterns demonstrated high reproducibility, and each displayed pattern is an average of the three replicates (see examples in Part S11, ESI†). To facilitate data interpretation, the patterns were compared in three separate sections, *i.e.*, *m*/*z* = 2000–15 000 (Fig. 3a/b/c-I), *m*/*z* = 15 000–29 000 (Fig. 3a/b/c-II) and *m*/*z* = 29 000–80 000 (Fig. 3a/b/c-III). Substantial improvements in pattern quality, both in terms of peak number and peak intensities, especially visible in the high mass range (*m*/*z* >15 000), were observed for all three bacteria samples when a photo-reactive TiO_2_-modified target plate was utilized. The total peak numbers (S/N > 3, counted with an open source mass spectrometry tool mMass, ; http://www.mmass.org/) were increased by 50–70%, *i.e.*, from 103 ± 1 to 174 ± 1 for *E. coli*, 87 ± 1 to 139 ± 2 for *P. aeruginosa* and 52 ± 1 to 88 ± 1 for *B. subtilis* (Fig. 3a/b/c-IV). The newly detected peaks, completely absent in the case of bare steel target plates, are mostly low intensity ones, corresponding to low-abundance or hardly-ionized bacterial components.

As the genomes of *B. subtilis* 168 have been completely sequenced, the 36 fingerprint peaks additionally detected using the TiO_2_-modified target plate (Fig. 3c) were analysed by a proteome database search. According to the search method provided by Fenselau *et al.*,^35^,^36^ 33 of the 36 peaks were tentatively assigned to proteins in the *B. subtilis* 168 proteome database (UniProtKB, proteome ID UP000001570) based on their *m*/*z* values. The list of assigned proteins is given in Part S12, ESI.† Each possibly matching protein was characterized by its subcellular location, isoelectric point (p*I*) and grand average value of hydropathicity (GRAVY). Results showed that these proteins mainly came from the cytoplasmic membrane and interior region (cytosol) of the bacteria cells (Part S13a, ESI†). The distribution of their p*I* values, varying between 3.72 and 10.73, indicated that TiO_2_ had no special preference in improving MALDI–TOF MS-based detection of basic (p*I* > 7) or acidic (p*I* < 7) proteins (Part S13b, ESI†). The distribution of the GRAVY index, normally used to evaluate the average protein hydrophobicity (GRAVY > 0) and hydrophilicity (GRAVY < 0), showed that most of these proteins were moderately hydrophilic (Part S13c, ESI†).

Overall, MALDI–TOF MS analysis of bacteria was promoted by TiO_2_, with significant improvement in both peak numbers and peak intensities of bacterial fingerprint patterns within a broad mass range. Attributed to its high photo-reactivity and photocatalytic bacteria disruption ability, TiO_2_ helped not only to break the cellular envelope structure, but also to enhance the desorption/ionization efficiency of intracellular components. By generating high quality bacterial fingerprint patterns, the TiO_2_-modified target plate could greatly boost the reliability of bacteria identification, which is based on fingerprint pattern matching. More importantly, it facilitates the extraction of more bacterial cellular information, and enables the detection of large molecular weight and low abundance bacterial components, especially those related to antimicrobial drug resistance, as discussed further.

### Detection of antimicrobial resistance-associated proteins from intact bacteria

**Fig. 4:**
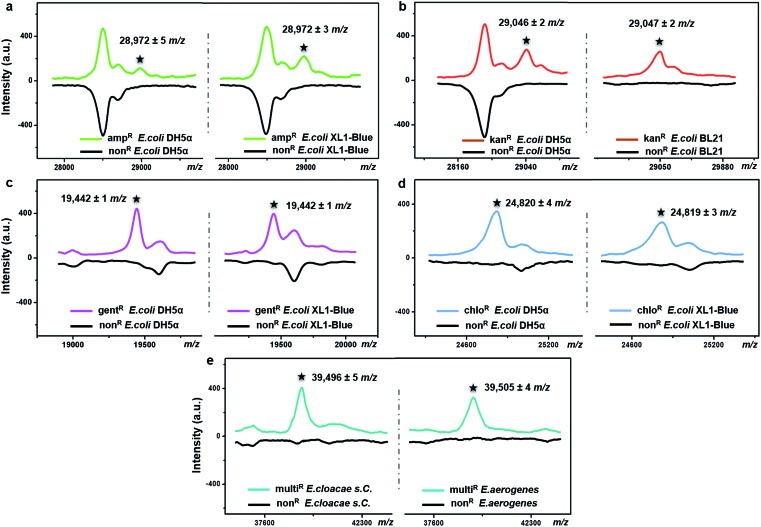
Detection of antimicrobial resistance-associated proteins from (a) ampicillin-resistant (amp^*R*^), (b) kanamycin-resistant (kan^*R*^), (c) gentamicin-resistant (gent^*R*^), and (d) chloramphenicol-resistant (chlo^*R*^) *E. coli* strains (two strains in each case) and (e) multidrug-resistant (multi^*R*^) *E. cloacae* s. *C.* and *E. aerogenes* by intact bacteria MALDI–TOF MS fingerprinting with TiO_2_ NP-modified target plates. Each pattern was averaged from three replicates. The number of bacterial cells on each sample spot was around 5 × 10^5^.

The possibility of antimicrobial resistance-associated protein detection by an intact bacteria MS fingerprinting approach was investigated with the TiO_2_-modified target plate. The detection was firstly conducted with bacteria samples that were modified by gene transfer. Corresponding plasmid DNAs, carrying specific resistance genes, were artificially transformed into recipient bacteria using recombinant techniques.^37^ Following this strategy, defined non-resistant (or antibiotic-susceptible) *E. coli* strains were equipped with the desired antimicrobial resistance, *i.e.*, resistance against ampicillin, kanamycin, gentamicin and chloramphenicol, respectively. MALDI–TOF MS fingerprint patterns of the resistant strains were measured within the mass range of *m*/*z* = 2000–80 000 and compared with those of non-resistant strains. To ensure result reliability, each type of resistance was repeatedly developed within two *E. coli* strains, *i.e.*, two DH5α, XL1-Blue or BL21. The MS results showed that resistance-associated proteins were successfully detected from all of these resistant strains (Fig. 4), as explained in further detail below.

Gene *bla*_TEM-1,_ encoding a TEM-1 β-lactamase, conferred resistance against ampicillin. With a molecular weight around 29 kDa, TEM-1 β-lactamase inactivates ampicillin by hydrolysis of the β-lactam ring in the ampicillin molecule.^38^ Compared to the ampicillin-susceptible *E. coli*, the ampicillin-resistant ones exhibited almost the same MALDI–TOF MS fingerprint patterns except for an additional peak at *m*/*z* = 28 972 ± 5 for strain DH5α and *m*/*z* = 28 972 ± 3 for strain XL1-Blue (Fig. 4a). This result coincides with a previous study, in which a special preparatory protein extraction was conducted before MS measurement.^38^ The resistance against kanamycin resulted from the expression of neomycin-kanamycin phosphotransferase type II (29 048 Da, UniProtKB-P00552), which inactivates kanamycin by phosphoryl transfer at its 3′-hydroxyl group.^39^ Using TiO_2_-modified target plates, this phosphotransferase was successfully detected in two kanamycin-resistant *E. coli* strains (at *m*/*z* = 29 046 ± 2 for strain DH5α, and *m*/*z* = 29 047 ± 2 for strain BL21), but not in their non-resistant counterparts (Fig. 4b). The resistance against gentamicin was conferred by gene *aacC1*, encoding gentamicin acetyltransferase I (19 442 Da, UniProtKB-P23181), which inactivates gentamicin by acetylating its 3-amino deoxystreptamine moiety.^40^ This protein was detected exclusively in the gentamicin-resistant *E. coli* at *m*/*z* = 19 442 ± 1 for both strains DH5α and XL1-Blue (Fig. 4c). The resistance against chloramphenicol was caused by the synthesis of chloramphenicol acetyltransferase (CAT, 24–26 kDa),^41^ which catalyses the transfer of an acetyl moiety from bacterial coenzyme A to the chloramphenicol molecules, and, therefore, results in antibiotic inactivation. Here, in contrast with non-resistant *E. coli*, a peak around *m*/*z* = 24 820 was clearly detected for the chloramphenicol-resistant *E. coli* (24 820 ± 4 for strain DH5α, 24 819 ± 3 for strain XL1-Blue), confirming the expression of CAT (Fig. 4d). For all of the above measurements, detection of each resistance protein showed high reproducibility for both tested *E. coli* strains. The allowed tolerance between the measured and the theoretical masses was 300 ppm, due to the limited resolving power of the MALDI–TOF MS instrument used.

To investigate the expression of the same resistance gene within different bacteria species, *Enterobacter cloacae* ssp. *cloacae* (*E. cloacae* s. *C.*) and *Enterobacter aerogenes* (*E. aerogenes*) were artificially transformed with an *ampC* gene encoding AmpC type β-lactamase (∼39.5 kDa)^42^,^43^ and measured with MALDI–TOF MS. After the gene transfer, both *E. cloacae* s. *C.* and *E. aerogenes* acquired resistance against 10 different β-lactam antibiotics, becoming multidrug resistant. Their detailed antimicrobial susceptibility profiles (measured with a bioMérieux VITEK 2 automated AST system based on an antimicrobial drug culture method) before and after the gene transfer are shown in Part S16, Tables S1–S4, ESI.† For the multidrug-resistant *E. cloacae* s. *C.* and *E. aerogenes*, the minimum inhibitory concentrations (MICs) of the 10 antibiotics varied from 16 to 128 μg mL^–1^. Compared to their non-resistant counterparts, the two resistant strains both exhibited an additional peak around *m*/*z* = 39 500 (*m*/*z* = 39 496 ± 5 and 39 505 ± 4, respectively) (Fig. 4e), confirming the expression of the AmpC type β-lactamase.

It should be mentioned that none of the above resistance-associated proteins were detectable when classic bare stainless steel plates were used (Part S14, ESI†), showing the importance of TiO_2_-modified target plates in bacteria analysis.

To further confirm the identity of the detected resistance-associated proteins, the antibiotic-resistant or non-resistant strains were analysed with a widely used proteomic approach. Bacteria cells were lysed in sodium dodecyl sulphate loading buffer, and the extracted proteins were separated by sodium dodecyl sulphate-polyacrylamide gel electrophoresis (SDS-PAGE) and subsequently identified by liquid chromatography-tandem mass spectrometry (LC-MS/MS). Taking gentamicin-resistant *E. coli* DH5α and kanamycin-resistant *E. coli* BL21 as examples, a protein band around 19 kDa or 29 kDa was clearly observed on their corresponding SDS-PAGE gel running lanes, but not observed for their non-resistant counterparts (Part S15, ESI†). Excision of the ∼19 kDa band from both gentamicin-resistant and non-resistant *E. coli* DH5α (as control) lanes, followed by digestion in trypsin, revealed the presence of 60 gentamicin acetyltransferase I exclusive unique peptides in the gentamicin-resistant strain, with 100% protein identification probability and 93% (164/177) amino acid coverage (Table S1 in Part S15, ESI†). The ∼29 kDa bands from kanamycin-resistant and non-resistant *E. coli* BL21 (as control) lanes were analysed in the same way, revealing the presence of 72 neomycin-kanamycin phosphotransferase type II exclusive unique peptides in the kanamycin-resistant strain, with 100% protein identification probability and 93% (246/264) amino acid coverage (Table S2 in Part S15, ESI†). The above results coincide with the MALDI–TOF MS intact bacteria fingerprinting results in Fig. 4, confirming the expression and identity of antibiotic resistance-associated proteins in corresponding resistant strains.

**Fig. 5:**
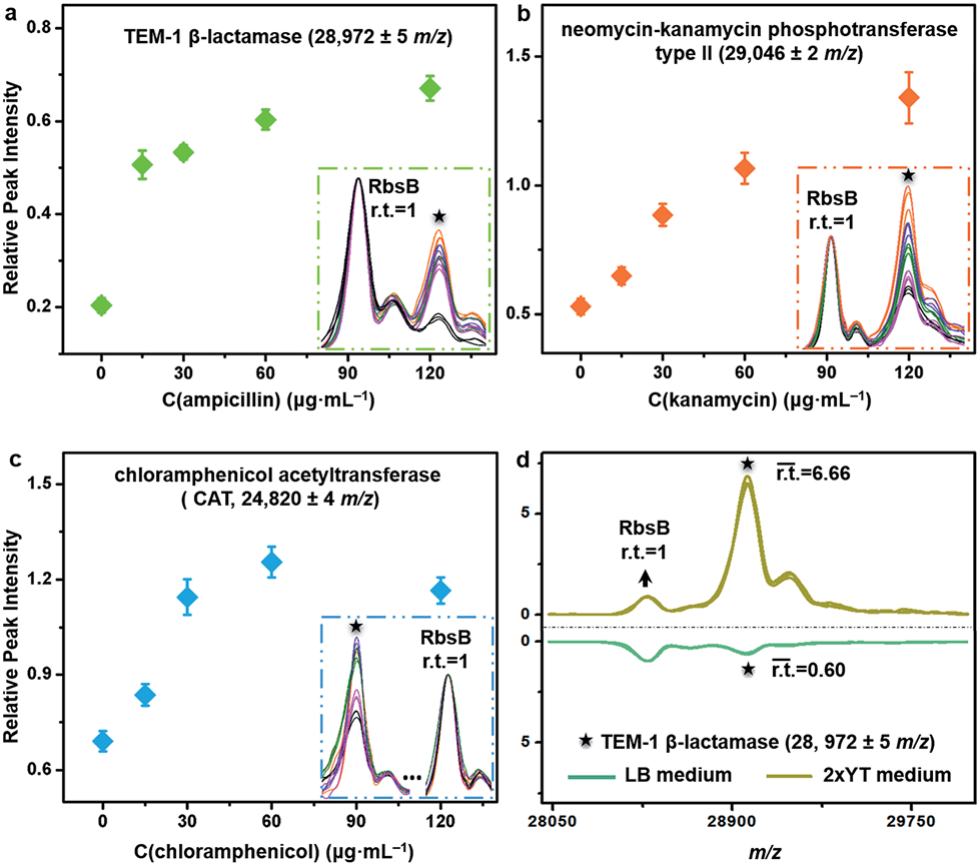
Measurement of variations in resistance protein expression levels within bacteria. Relative MS peak intensities of (a) TEM-1 β-lactamase, (b) neomycin-kanamycin phosphotransferase type II or (c) CAT detected from ampicillin-resistant, kanamycin-resistant or chloramphenicol-resistant *E. coli* DH5α that were cultured with LB medium containing ampicillin, kanamycin or chloramphenicol, respectively. The concentration of each antibiotic was set as 0, 15, 30, 60 and 120 μg mL^–1^. Corresponding MALDI–TOF MS patterns of related peaks (with overlapping of three replicates for each test) are shown in the insert graphs. (d) MALDI–TOF MS patterns in the mass range of *m*/*z* = 28 000–30 000 (with overlapping of three replicates) of ampicillin-resistant *E. coli* DH5α that was cultured with LB or 2xYT medium containing 60 μg mL^–1^ of ampicillin. Relative intensities (r.t.) of all peaks were calculated using a signal from protein RbsB as an internal intensity standard (r.t._RbsB_ = 1). The data were obtained by intact bacteria MALDI–TOF MS fingerprinting using TiO_2_ NP-modified target plates. The number of bacterial cells on each sample spot was around 5 × 10^5^.

The described TiO_2_-facilitated MALDI–TOF MS approach can also quickly sense the variations in resistance genes’ expression levels within bacteria. To demonstrate this, antibiotic-resistant bacteria were cultured in Luria-Bertani (LB) medium that contained different concentrations of corresponding antibiotics. A gradual increase of a given antibiotic concentration brings a proportionally higher selection pressure to the bacterial cells. As a response, bacterial cells modulate the resistance genes’ expression level to increase the synthesis of resistance proteins for survival.^44^ Such kinds of change were measured for ampicillin-, kanamycin- and chloramphenicol-resistant *E. coli* DH5α by comparing the relative peak intensities (r.t.) of the corresponding resistance proteins in the MALDI–TOF MS fingerprint patterns (Fig. 5a–c). The r.t. of the resistance proteins (*i.e.*, TEM-1 β-lactamase at *m*/*z* = 28 972 ± 5, neomycin-kanamycin phosphotransferase type II at *m*/*z* = 29 046 ± 2 and CAT at *m*/*z* = 24 820 ± 4) were calculated using signals from *E. coli* DH5α d-ribose-binding periplasmic protein (RbsB, ∼28.5 kDa)^45^ as an internal intensity standard (r.t._RbsB_ = 1). For all three proteins, their r.t. increased with an increase in corresponding antibiotic concentration. These data confirm that higher levels of antibiotic resistance would accompany higher expression levels of resistance proteins and consequently higher r.t. values of the corresponding MS peaks. For the chloramphenicol-resistant strain, however, the r.t. of CAT decreased when the chloramphenicol concentration reached 120 μg mL^–1^ (Fig. 5c). Probably, this concentration was already too high and started to negatively affect the bacterial physiological state. In addition to the antibiotics present, the type of culture medium can also affect the expression level of resistance proteins. Synthesis of resistance proteins to fight against antibiotics is an energy-consuming process, which can be positively influenced by the use of nutritionally rich growth medium.^46^ To observe this effect, ampicillin-resistant *E. coli* DH5α was cultured in different growth media containing a fixed concentration (60 μg mL^–1^) of ampicillin. The corresponding MALDI–TOF MS fingerprint patterns indicated that 2xYT medium, specifically rich in amino acids and peptides, favoured the up-regulation of gene *bla*_TEM-1_ expression. In particular, when the growth medium was changed from LB to 2xYT, the averaged r.t. of TEM-1 β-lactamase increased from 0.60 to 6.66 (r.t._RbsB_ = 1) (Fig. 5d).

### Simultaneous bacteria identification and antimicrobial resistance-associated protein detection with clinical pathogens

The proposed method can be used for antimicrobial resistance protein detection whilst performing bacteria species identification. The feasibility was explored with three clinical pathogens: extended-spectrum β-lactamase-producing *E. coli* (ESBL-*E. coli*), multidrug-resistant *Pseudomonas aeruginosa* (MDR-*P. aeruginosa*) and methicillin-resistant *Staphylococcus aureus* (MRSA).

**Fig. 6:**
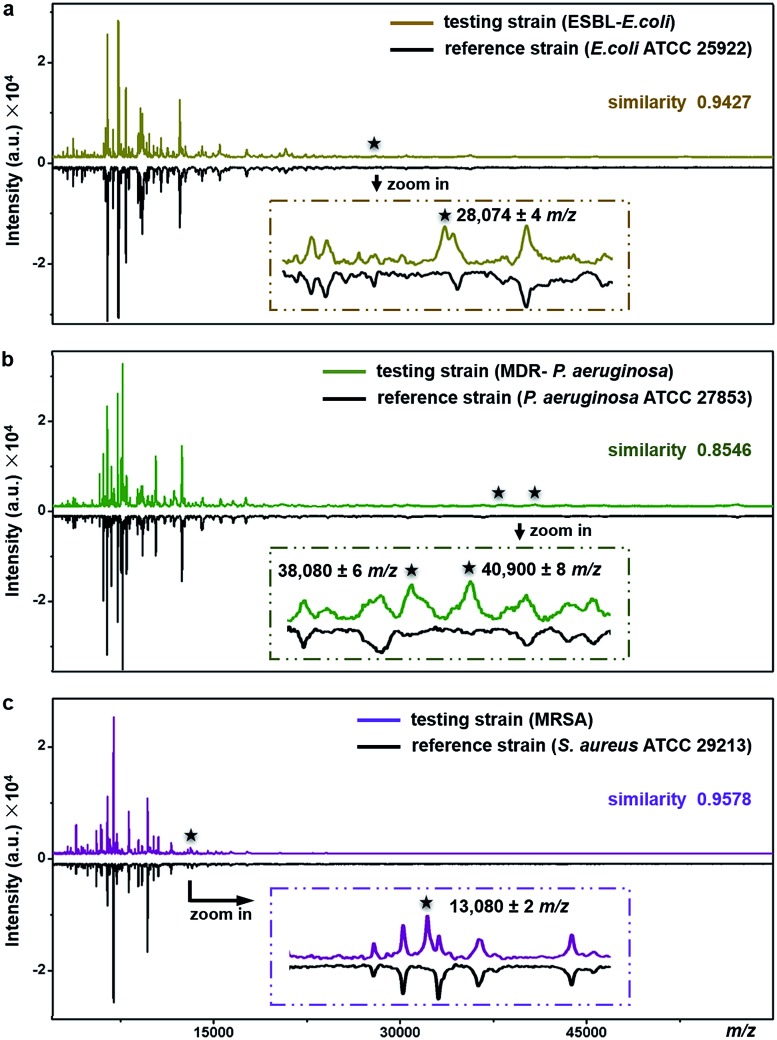
Simultaneous species identification and antimicrobial resistance-associated protein detection of clinical pathogens, *i.e.*, (a) ESBL-*E. coli*, (b) MDR-*P. aeruginosa* and (c) MRSA, by comparing their MS fingerprint patterns with those of reference strains (*i.e.*, *E. coli* ATCC25922, *P. aeruginosa* ATCC 27853 and *S. aureus* ATCC29213). The data were obtained by intact bacteria MALDI–TOF MS fingerprinting using TiO_2_ NP-modified target plates. Each pattern was averaged from three replicates. The number of bacterial cells on each sample spot was around 5 × 10^5^.

ESBL, first reported in Germany in 1983, confers resistance to a broad spectrum of β-lactam antibiotics.^47^ Worldwide emergence of ESBL-*E. coli* raises serious therapeutic problems. Resistance in the ESBL-*E. coli* tested here was conferred by the expression of CTX-M type β-lactamase (∼28 kDa).^48^*E. coli* ATCC25922, a strain without such kind of resistance, was used as the reference for species identification and resistance protein detection of the testing strain (ESBL-*E. coli*). Detailed antimicrobial susceptibility profiles of the two strains, measured with a bioMérieux VITEK 2 automated AST system, are shown in Part S16, Tables S5 and S6, ESI.† For the testing strain, the MICs of the corresponding antibiotics were 4–320 μg mL^–1^ (Table S6†). The averaged MALDI–TOF MS fingerprint patterns of the two strains are displayed in Fig. 6a. The similarity score between the two patterns was calculated using a public bacteria identification platform, BacteriaMS, with a cosine correlation algorithm (; http://bacteriams.fudan.edu.cn/#/). This algorithm gives the maximum score as 1.0. Here, with the pattern similarity as high as 0.9427, the testing strain was identified to be the same species as the reference one, *i.e.*,*E. coli*. The two strains shared almost all MS peaks (S/N > 3, r.i. > 0.1%) in the mass range of *m*/*z* = 10 000–80 000, except for a peak at *m*/*z* = 28 074 ± 4 only detected for the testing strain (Fig. 6a, zoom-in). The appearance of this peak most probably results from the expression of CTX-M type β-lactamase. Therefore, together with the species identification, the CTX-M type ESBL resistance was recognized in the testing strain.

Similarly, the MDR-*P. aeruginosa* and MRSA were also identified at the species level by comparison of their fingerprint patterns with those of corresponding reference strains (*i.e.*, *P. aeruginosa* ATCC 27853 and *S. aureus* ATCC 29213), resulting in pattern similarity scores of 0.8546 and 0.9578, respectively (Fig. 6b and c). Simultaneously, two mass spectral peaks, at *m*/*z* = 38 080 ± 6 and *m*/*z* = 40 900 ± 8, were exclusively observed from the MDR-*P. aeruginosa* (Fig. 6b, zoom-in). They most likely come from efflux pump proteins MexA (∼38 kDa)^49^,^50^ and MexX (40.9 kDa, UniProtKB-Q9ZNG9), which confer the multidrug resistance of the MDR-*P. aeruginosa*. These two proteins are involved in the extrusion of β-lactam antibiotics (*e.g.*, tazobactam, ceftazidime, and cefepime) and aminoglycosides (*e.g.*, amikacin, gentamicin, netilmicin, and tobramycin) from within bacteria cells into the external environment.^51^ Meanwhile, antimicrobial resistance in MRSA, one of the most common multidrug resistant pathogens, arises from the expression of gene *mecA*, which causes the alteration of penicillin binding protein (PBP) and triggers the expression of its alternative, *i.e.*, PBP 2a (∼78 kDa). PBP 2a has a low affinity for most β-lactam antibiotics including methicillin, thereby making bacteria resistant against them.^52^ According to previous studies, a characteristic fragment of PBP 2a (∼13 kDa) can be detected for MRSA by a proteomics-based method.^53^–^55^ In the present work, a peak at *m*/*z* = 13 080 ± 2 was exclusively detected for the MRSA strain (Fig. 6c, zoom-in), which could come from the PBP 2a fragment. To confirm this assumption, two more MRSA strains were tested and the peaks around 13 kDa (at *m*/*z* = 13 083 ± 3 and *m*/*z* = 13 081 ± 4, respectively) were repeatedly detected, as shown in Part S17, ESI.† The detailed antimicrobial susceptibility profiles of MDR-*P. aeruginosa*, MRSA and their reference strains are shown in Part S16, Tables S7–S12, ESI.† As shown in these profiles, antibiotic MICs for the resistant strains were measured as 0.5–8 μg mL^–1^.

Herein, antimicrobial resistance-associated proteins were successfully detected directly in intact bacteria without any sample pre-treatment, by TiO_2_-facilitated MALDI–TOF MS. The developed approach showed feasibility for both Gram-negative and Gram-positive bacteria species bearing different types of antimicrobial resistance. Each of the resistance proteins were specifically detected from the corresponding antibiotic-resistant strains, not from the non-resistant reference strains or the strains resistant to other antibiotics. For certain resistance types tested in this work, it was also shown that higher levels of antibiotic resistance could accompany higher expression levels of the resistance proteins. Sensing of the expression level variations was proven possible through direct readout of the relative intensities of the corresponding MS peaks.

The described method performed resistance protein recognition according to their *m*/*z* values. Due to the limited resolving power of current MALDI–TOF MS instruments, it would be difficult to distinguish closely-related protein isoforms with quite similar molecular weights like TEM-1, TEM-2 and TEM-3 β-lactamases that differ only in a few amino acid substitutions. This is a drawback for MALDI–TOF MS-based analysis of proteins in comparison with nucleic acid-based molecular detection of the related genes or proteomics-based approaches. In this work, it has been confirmed that the expression levels of resistance proteins directly determine their MS peak appearances in bacterial fingerprint patterns. As intact bacteria are analysed directly without preparatory protein extraction, enrichment or selective separation, the proposed method could lack some sensitivity when the resistance proteins are expressed at a very low level. Here, the method was shown to be sensitive enough for resistant strains with antibiotic MICs as low as a few microgram per millilitre when 1 uL of the bacteria sample (∼5 × 10^5^ cells) was measured.

Nonetheless, compared to existing methods for resistance gene or protein detection such as nucleic acid-based molecular techniques or proteomics-based approaches, the proposed MALDI–TOF MS-based method has clear advantages of simplicity and rapidity of sample preparation, measurement protocol and data analysis. It is a useful procedure for quick discrimination of antimicrobial-resistant bacteria strains from their non-resistant counterparts, as well as a fast method for the initial determination of resistance mechanisms and prediction of antibiotic types or classes that the strains could be resistant to.

## Conclusions

In this work, intact bacteria MALDI–TOF MS analysis was improved by TiO_2_ due to its ability to photo-catalytically destroy bacterial envelopes and to facilitate analyte desorption/ionization. Impressive improvement in detection sensitivity and working mass range was achieved, pushing the current limits of the bacteria MALDI–TOF MS fingerprinting approach. Accordingly, antimicrobial resistance-associated proteins, especially those larger than 15 kDa, were successfully detected from intact bacteria by the direct readout of the corresponding MS peaks from the fingerprint patterns, together with a rapid sensing of their expression level variations. With the potential of simultaneous species identification and antimicrobial resistance analysis, the TiO_2_-facilitated MALDI–TOF MS opens new avenues for bacteria analysis.

## Conflicts of interest

There are no conflicts to declare.

## Supplementary Material

Supplementary information
