# Supplementary material for: Detection of antimicrobial resistance-associated proteins by titanium dioxide-facilitated intact bacteria mass spectrometry
Source: Chem Sci. 2018 Jan 18;9(8):2212–21. doi: 10.1039/c7sc04089j (PMC5897883; doi:10.1039/c7sc04089j)
Supplement: Supplementary file 1 [file SC-009-C7SC04089J-s001.pdf]

Electronic Supplementary Material (ESI) for Chemical Science.
This journal is © The Royal Society of Chemistry 2018

Electronic Supplementary Information (ESI) for:

Detection of antimicrobial resistance-associated proteins by titanium dioxide-facilitated intact bacteria mass spectrometry

Yingdi Zhu,[a] Natalia Gasilova,[a,b] Milica Jović,[a] Liang Qiao,[c] Baohong Liu,[c] Lysiane Tissières Lovey,[d] Horst Pick[e] and Hubert H. Girault[a]*

a. Laboratoire d'Electrochimie Physique et Analytique, École Polytechnique Fédérale de Lausanne, Rue de l'industrie 17, CH-1951 Sion, Switzerland.

b. ISIC-GE-VS, École Polytechnique Fédérale de Lausanne, Rue de l'industrie 17, CH-1951 Sion, Switzerland.

c. Department of Chemistry, Fudan University, Handan Road 220, 200433 Shanghai, China.

d. ICH, Hôpital du Valais, Avenue du Grand Champsec 86, CH-1951 Sion, Switzerland.

e. Laboratoire de Chimie Biophysique des Macromolécules, École Polytechnique Fédérale de Lausanne, CH-1015 Lausanne, Switzerland.

*Correspondence should be addressed to H.H.G. (hubert.girault@epfl.ch)

CONTENT

Experimental ....**page 3**

Supplementary Results and Discussion

S1. Comparison of different matrices for intact bacteria MALDI-TOF MS fingerprinting ....**page 9**

S2. Surface roughness profiles of a classic bare and a TiO_2-modified MALDI target plates..............**page 11**

S3. Comparison of water contact angle on a classic bare and a TiO_2-modified MALDI target plates..............**page 12**

S4. UV-visible absorption spectrum of TiO_2 NPs..............**page 13**

S5. TiO_2-triggered photocatalytic reactions related to the generation of reactive oxygen species..............**page 14**

S6. Influence of reactive oxygen species scavengers on bacterial fingerprinting patterns..............**page 15**

S7. MALDI-TOF MS patterns of bacterial protein extracts ....**page 16**

S8. Comparison of the bare and TiO_2-modified target plates for the detection of standard protein mixture samples by MALDI-TOF MS..............**page 17**

S9. MALDI-TOF MS patterns of intact bacteria using target plates modified with different nanomaterials..............**page 18**

S10. MALDI-TOF MS spectra of standard protein mixtures using target plates modified with different nanomaterials..............**page 19**

S11. An example of bacterial MALDI-TOF MS fingerprint patterns from three replicates and the averaged pattern..............**page 20**

S12. A list of proteome database search result ....**page 21**

S13. Characterization of the newly detected MS peaks from *B. subtilis* 168 with TiO_2-modified target plates by proteome database search..............**page 23**

S14. Unsuccessful detection of antimicrobial resistance-associated proteins by using classic bare stainless steel target plates..............**Page 24**

S15. LC-MS/MS analysis of SDS-PAGE gel bands..............**Page 25**

S16. Lists of antimicrobial susceptibility profiles ....**page 31**

S17. Detection of antimicrobial-resistance associated proteins from two more methicillin-resistant *Staphylococcus aureus* strains..............**Page 37**

Reference..............**page 38**

Experimental

Materials and reagents

TiO_2 nanopowder (Aeroxide® P25, 21 nm primary particle size, ≥ 99.5% trace metals basis), α-cyano-4-hydroxycinnamic acid (CHCA) (matrix substance for MALDI-MS, ≥ 99.0% HPLC), 2,5-dihydroxybenzoic acid (DHB) (matrix substance for MALDI-MS, ≥ 99.0% HPLC), sinapinic acid (matrix substance for MALDI-MS, ≥ 99.0% T), *trans*-ferulic acid (matrix substance for MALDI-MS, ≥ 99.0% HPLC), ethanol (≥ 99.8%, GC) and formic acid (ACS reagent, ≥ 96.0%) were bought from Sigma-Aldrich (St. Gallen, Switzerland). Acetic acid (glacial, 100%, EMSURE® ACS) was bought from Merck Millipore (Darmstadt, Germany). Acetonitrile (HPLC grade) was purchased from Aventor Performance Materials (Center Valley, PA, USA). Trifluoroacetic acid (99.0%, extra pure) was obtained from Acros Organics (New Jersey, USA). Deionized (DI) water (18.2 MΩ cm) was purified by an alpha Q Millipore system (Zug, Switzerland), and used in all aqueous solutions. Luria-Bertani (powder microbial growth medium), 2xYT medium (powder microbial growth medium), ampicillin (anhydrous basis, 96.0-100.5%), kanamycin sulfate salt (USP grade), gentamycin sulfate (USP grade) and chloramphenicol (USP grade) were purchased from Sigma-Aldrich (St. Gallen, Switzerland).

Fabrication of TiO_2 NPs-modified MALDI target plates

TiO_2 NPs aqueous suspension was prepared according to a previously reported method.[1] Briefly, 1 g of P25 TiO_2 nanopowder was beforehand heated at 300 °C for 2 h and then separated in a mortar for 3 h. During the separation process, 1 mL of 10% acetic acid aqueous solution was added drop by drop to keep the nanopowder wet. The separated nanoparticles were suspended in an aqueous solution of ethanol (89%, V/V) to reach a concentration of 100 mg·mL^{-1}, followed by ultra-sonication for 1 h. The suspension was then diluted 25 times with DI water to reach a final concentration of 4 mg·mL^{-1}. Such obtained TiO_2 NPs suspension was stored at 4 °C and was stable for four to six months.

The TiO_2 NPs-modified MALDI target plates were prepared by depositing the TiO_2 suspension onto the spots of a classic bare stainless steel target plate (here, commercial MSP 96 ground steel target, Bruker Daltonics). The suspension was deposited by drop casting or by dispenser in the following manner: 2 µL of the suspension was firstly dropped onto each spot and air-dried for ~10 min; thereafter, another 2 µL of the suspension was dropped to cover the previous one and again air-dried for ~10 min. Alternatively, the TiO_2 suspension could also be dropped as an array of spots onto a piece of stainless steel foil (20 µm thick), which was afterwards affixed onto a commercial bare target plate before MALDI-TOF MS measurement. The dried target plate or steel foil was heated at 400 °C to sinter the nanoparticles. The heating process was accomplished with a three-step automatic program: raising temperature from 25 to 400 °C within

1h, keeping the temperature at 400 °C for 1h, cooling down to 25 °C within 4 h. Alternatively, instead of thermal heating at 400 °C, the sintering process could also be completed by photonic curing, which takes only a few miliseconds. The photonic curing was conducted using high intensity light pulses from a xenon flash lamp provided by a PulseForge 1300 photonic curing station (NovaCentrix, USA). The curing parameters were set as: 5 pulses exposure, 450 V bank voltage, 1 ms pulse duration. Through sintering, a stable layer of TiO_2 NPs was formed and firmly attached on the steel surface. The sintering process did not change the crystalline phase of TiO_2, which was kept as the mixed rutile (110) and anatase (101) (mainly) phase. Before the usage, prepared TiO_2-modified target plate or steel foil pieces were stored in a clean and dry room temperature environment.

Incubation of bacteria

Escherichia coli strains DH5α, XL1-Blue, BL21 and *Bacillus subtilis* strain 168 were grown as pre-cultures in Luria-Bertani (LB) medium at 37 °C for 6 h with continuous shaking at 200 rpm. 100 µL of each pre-culture was added into 3 mL of LB and incubated overnight at 37 °C with continuous shaking at 200 rpm, and the obtained fresh cultures were afterwards analyzed with MALDI-TOF MS.

Non-resistant *Enterobacter cloacae ssp. cloacae*, multidrug-resistant *Enterobacter cloacae ssp. cloacae* (carrying resistance gene *AmpC*), non-resistant *Enterobacter aerogenes*, multidrug-resistant *Enterobacter aerogenes* (carrying resistance gene *AmpC*), *Escherichia coli* ATCC 25922, CTX-M type extended-spectrum β-lactamase-producing *Escherichia coli*, *Pseudomonas aeruginosa* ATCC 27853, multidrug-resistant *Pseudomonas aeruginosa*, *Staphylococcus aureus* ATCC 29213 and methicillin-resistant *Staphylococcus aureus*, as well as the detailed information about antimicrobial resistance in each resistant strain, were provided by a local hospital (Hôpital du Valais, Sion, Switzerland). Their detailed antimicrobial susceptibility profiles were measured using a bioMérieux VITEK 2 automated AST system, based on antimicrobial drugs culture method. These strains were directly analyzed with MALDI-TOF MS when they were obtained.

Concentrations of bacterial cells in bacteria samples were determined by measuring the optical density at 600 nm (OD_{600nm}). 1.0 OD_{600nm} corresponds to ~8×10^8 cells·mL^{-1}.

Transfer of resistance genes into bacteria

Plasmid DNAs carrying a specific antimicrobial resistance gene were transformed into two *Escherichia coli* (*E. coli*)

strains, *i.e.* two of DH5α, XL1-Blue or BL21, according to the protocol provided by Sambrook and Russel.[2] The plasmids utilized were: pBluescriptIISK(+) carrying resistance against ampicillin (Stratagene, California, USA), pEGFP-N1 carrying resistance against kanamycin (Clontech, California, USA), pEN_TmiRc3 carrying resistance against gentamycin (addgene, Massachusetts, USA) and pOFXT7-2 carrying resistance against chloramphenicol (donated as a gift from University of Lausanne, Lausanne, Switzerland). Specifically, 1.5 mL of each *E. coli* pre-culture was overnight incubated in 50 mL of LB medium at 37 °C with continuous shaking at 200 rpm. The culture was transferred into an ice-cold polypropylene tube and cooled on ice for 10 min. *E. coli* cells were separated from the growth medium by centrifugation at 2700 × *g* for 10 min at 4 °C. After thoroughly removing the growth medium, the cell pellet was suspended in 30 mL of ice-cold $MgCl_2$-$CaCl_2$ solution (80 mM $MgCl_2$, 20 mM $CaCl_2$) by gentle vortexing. The cells were again collected by centrifugation at 2,700 × *g* for 10 min at 4 °C, and gently resuspended in 2 mL of ice-cold 0.1 M $CaCl_2$. The resulting competent *E. coli* were either directly used for transformation as described below or dispensed into aliquots and stored at -70 °C.

For transformation, 200 µL of above obtained competent *E. coli* cells were mixed with 100 ng of purified plasmid DNAs that carried a specific resistance gene. The mixture was incubated on ice for 30 min, and then applied with a heat shock for exactly 90 s in a 42 °C water bath. Thereafter, the mixture was rapidly transferred into an ice bath and chilled for 2 min. After adding 800 µL of LB medium, the mixture was incubated for 45 min in a 37 °C water bath to let the bacteria recover and express the antibiotics resistance marker encoded by the plasmid. The obtained bacteria were spread on selective agar plates containing 20 mM $MgSO_4$ and appropriate antibiotic. Single colonies were picked up and added into 2 mL of LB for overnight incubation at 37 °C with continuously shaking at 200 rpm. The resulting ampicillin-resistant, kanamycin-resistant, gentamicin-resistant or chloramphenicol-resistant *E. coli* strains were either directly analyzed with MALDI-TOF MS or incubated with antibiotics as described below.

Experimentally controlling the resistance gene expression level within *E. coli*

In order to experimentally control the resistance gene expression level within bacteria, the above obtained ampicillin-resistant, kanamycin-resistant and chloramphenicol-resistant *E. coli* DH5α cultures were diluted 100 times with LB medium containing gradually increased concentration of corresponding antibiotic (*i.e.* ampicillin, kanamycin and chloramphenicol, respectively). The concentrations of each antibiotic were selected as 0, 15, 30, 60 and 120 $\mu g \cdot mL^{-1}$. The maximum concentration value (120 $\mu g \cdot mL^{-1}$) was lower than the minimum inhibitory concentration to allow the proliferation of bacterial cells.

To investigate the influence of growth medium, ampicillin-resistant *E. coli* DH5α were diluted 100 times with different growth media (LB medium, or 2xYT medium) containing 60 $\mu g \cdot mL^{-1}$ of ampicillin.

All of the above mixtures were incubated overnight at 37 °C with continuously shaking at 200 rpm. The obtained fresh cultures were afterwards analyzed with MALDI-TOF MS.

MALDI-TOF MS fingerprinting of intact bacteria with TiO_2 NPs-modified target plates or classic bare target plates

For each bacteria sample, bacterial cells were harvested from the growth medium by centrifugation at 7,500 × *g* for 3 min, then washed with DI water for two times. The cell pellet was finally suspended in DI water to reach a concentration of ~5 × 10^8 cells·mL^{-1}. The obtained intact bacteria aqueous solutions were deposited onto the spots of a TiO_2-modified target plate or a classic bare target plate (1 µL for each spot) by drop casting, then air-dried (~5 min). Each test was performed in triplicates by depositing the bacteria solution on three spots. Sinapinic acid matrix (15 mg·mL^{-1} in 50/49.9/0.1% acetonitrile/water/ trifluoroacetic acid) was dropped onto the dried sample spots to overlay the bacterial cells (1 µL for each spot). Bacteria/sinapinic acid co-crystals were formed after air-drying for ~5 min. Thereafter, the target plate was loaded into a MALDI-TOF MS instrument for measurement.

In order to compare the behavior of different matrices for intact bacteria fingerprinting, the dried sample spots with *E. coli* DH5α cells were overlaid with four different matrices: CHCA (10 mg·mL^{-1} in 50/49.9/0.1% acetonitrile/water/trifluoroacetic acid), DHB (10 mg·mL^{-1} in 50/49.9/0.1% acetonitrile/water/trifluoroacetic acid), sinapinic acid (15 mg·mL^{-1} in 50/49.9/0.1% acetonitrile/water/trifluoroacetic acid), *trans*-ferulic acid (12.5 mg·mL^{-1} in 17/33/50% formic acid/acetonitrile/ water) (1 µL for each spot), respectively.

Throughout the present work, all MALDI-TOF MS measurements were conducted with aqueous solutions of intact whole bacteria, except that protein extracts from *E. coli* DH5α was utilized for the characterization of TiO_2-modified target plates in **Part S7**. This protein extracts was prepared according to the typical ethanol/formic acid/acetonitrile extraction protocol, which is described in the Bruker MALDI Biotyper 3.0 user manual (2011). Briefly, *E. coli* DH5α cells were harvested from 1 mL of fresh culture by centrifugation at 7,500 × *g* for 3 min and washed two times with DI water. The cell pellet was suspended in 300 µL of DI water, followed by adding 900 µL of ethanol and vortexing thoroughly. The water and ethanol was completely removed by centrifugation at 8,500 × *g* for two times (2 min for each time) and air-drying for ~30 min. The obtained cell pellet was resuspended in 50 µL of 70/30% formic acid/water. The mixture was vortexed thoroughly and let stand for 5 min, followed by adding 50 µL of acetonitrile. The final mixture was vortexed thoroughly for another 5 min. Thus, intracellular proteins were extracted into the solvent. After centrifugation at 8,500 × *g* for 2 min, the supernatant was pipetted onto three spots of a TiO_2-modified target plate and a bare target plate (1 µL for each spot). After air-drying for ~5 min, each sample spot was overlaid with 1 µL of sinapinic acid matrix (15

mg·mL^{-1} in 50/49.9/0.1% acetonitrile/water/trifluoroacetic acid) and again air-dried (~5 min).

All measurements were conducted with Bruker MicroFlex LRF MALDI-TOF MS instrument (Bremen, Germany) under linear positive mode at 20 kV accelerating voltage. Instrumental parameters were set as: mass range 2,000-80,000 *m/z*, laser intensity 70%, laser attenuator with 30% offset and 40% range, 500 laser shots accumulation for each spot, 20.0 Hz laser frequency, 20× detector gain, suppress up to 1000 Da, 350 ns pulsed ion extraction.

At the beginning of each measurement, mass calibration was conducted with a calibration sample containing 1 mg·mL^{-1} of cytochrome c (*m/z*[M+2H]$^{2+}$=6181.05000, *m/z*[M+H]$^{+}$=12360.97000), 1 mg·mL^{-1} of myoglobin (*m/z*[M+2H]$^{2+}$= 8476.66000, *m/z*[M+H]$^{+}$=16952.31000) and 1 mg·mL^{-1} of protein A (*m/z*[M+2H]$^{2+}$=22307.00000, *m/z*[M+H]$^{+}$=44613.00000).

All experiments related to bacteria and antibiotics were conducted in a biosafety level 1 or 2 (P1 or P2) laboratory. Experimental supplies including centrifuge tubes and micropipette tips were disposable and sterile. All wastes were autoclaved and disposed properly according to the safety guidelines. Instruments, facilities and benches were wiped with 70/30% ethanol/water when experimental activities were finished.

Data analysis

Mass spectral fingerprint patterns of bacteria were visually compared using the mMass Open Source Mass Spectrometry Tool (http://www.mmass.org/). To facilitate data interpretation, the patterns were compared in three separate sections, *i.e.* 2,000-15,000 *m/z*, 15,000-29,000 *m/z* and 29,000-80,000 *m/z*. The peak numbers in each section were automatically countered by using the mMass tool, with "peak picking" parameters set as: *S/N* threshold 3.0, relative intensity threshold 2.0% for 2,000-15,000 *m/z* and 0.1% for 15,000-80,000 *m/z*, apply smoothing, remove shoulder peaks.

The 36 fingerprint peaks newly detected from *Bacillus subtilis* (strain 168) using a TiO_2 NPs-modified target plate in contrast with a classic bare one were analyzed by proteome database search, according to the method provided by Fenselau *et al.*[3, 4] The search was conducted against *Bacillus subtilis* 168 proteome database (UniProtKB, proteome ID UP000001570) on UniProtKB/Swiss-Prot/TrEMBL database platform (http://www.uniprot.org/). The primary search parameter was the experimental *m/z* value of each peak (averaged value from three replicates). During the search process, the mass window was selected as 300 ppm (± $m/z_{\text{experimental}}$ × 0.03%) for the mass range of 2,000-80,000 *m/z*. Information about subcellular location of each tentatively assigned protein was directly obtained from the database. Their isoelectric point (p*I*) and grand average value of hydropathicity (GRAVY) was obtained using the

ExPASy-ProtParam computation tool (http://web.expasy.org/protparam/) by entering the UniProtKB/ Swiss-Prot/TrEMBL accession number of each protein and following the links to "Compute parameters" and later "Submit".

Fingerprint patterns of a testing bacteria and a reference bacteria were mathematically compared using a public bacteria identification service platform Bacteria MS (http://bacteriams.fudan.edu.cn/#/). Raw data files (in '.txt' format) of three replicated patterns from a testing and a reference bacteria were uploaded onto this platform. The system then automatically averaged the three replicates, and the averaged patterns of the testing and the reference bacteria were compared by choosing the cosine correlation method. Information including identical peaks, different peaks, their normalized intensities and the similarity score of the two patterns were thus obtained. As illustrated in our previous work,[5] with the cosine correlation algorithm, the similarity score between two mass spectra (*i* and *j*) was defined as:

$$\cos = \frac{\vec{y_i} \cdot \vec{y_j}}{\left|\vec{Y_i}\right| \cdot \left|\vec{Y_j}\right|} = \frac{\sum_{k=1}^{l} y_{ik} y_{jk}}{\sqrt{\sum_{t=1}^{n_i} Y_{it}^2} \cdot \sqrt{\sum_{t=1}^{n_j} Y_{jt}^2}}$$

where *y* is the normalized intensity of a peak appearing in both spectra *i* and *j* (identical peak), *l* is the number of identical peaks in the two spectra, *Y* is the normalized intensity of a peak appearing in a spectrum, *n* is the number of peaks in a spectrum. Only peaks with $S/N \geq 3$ were taken into account. Peaks appearing in different spectra with $\Delta(m/z)/(m/z) \leq 300$ ppm were considered as identical peaks. This 300 ppm tolerance was chosen according to the resolving power of linear mode TOF analysis. It has been demonstrated that a similarity score of ≥ 0.8 ensures a successful identification at the species level.

Supplementary Results and Discussion

S1. Comparison of different matrices for intact bacteria MALDI-TOF MS fingerprinting

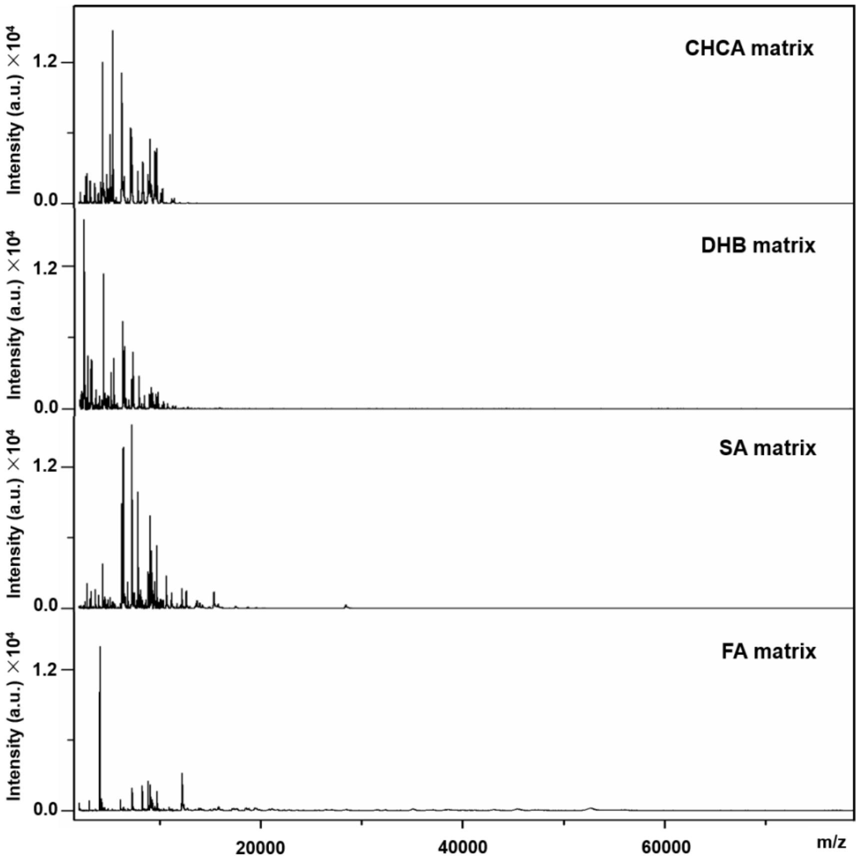

Figure S1 Comparison of different matrices for intact bacteria MALDI-TOF MS fingerprinting. The patterns were generated from intact *Escherichia coli* (strain DH5α) in the mass range of 2,000-80,000 *m/z* with the utilization of four matrices: α-cyano-4-hydroxycinnamic acid (CHCA), 2,5-dihydroxybenzoic acid (DHB), sinapinic acid (SA) and *trans*-ferulic acid (FA). A classic bare stainless steel MALDI target plate was employed. Number of bacterial cells on each spot was around 5×10^5. Each pattern was averaged from three replicates.

As shown in **Fig. S1**. CHCA, DHB and SA matrices exhibited high detection sensitivity in the typical mass range for bacteria identification (2,000-15,000 *m/z*). They also provided satisfying reproducibility, with pattern similarity scores > 0.99 for three replicates. The similarity scores between MS patterns were calculated using a public bacteria identification service platform BacteriaMS (http://bacteriams.fudan.edu.cn/#/) by choosing the cosine correlation algorithm, which gives the maximum score as 1.0. Proteins detectable with CHCA and DHB were smaller than ~15,000 Da, and the ones detectable with SA were a little larger. Meanwhile, FA matrix gave more peaks in the mass range of

15,000-80,000 *m/z*, but the sensitivity in 2,000-15,000 *m/z* was drastically decreased together with the reproducibility, giving pattern similarity scores lower than 0.5 for three replicates. Thus, SA matrix was selected for further study in this work, due to its ability to generate MS patterns with high quality within 2,000-15,000 *m/z* and higher for detection of large bacterial components.

S2. Surface roughness profiles of a classic bare and a TiO_2-modified MALDI target plates

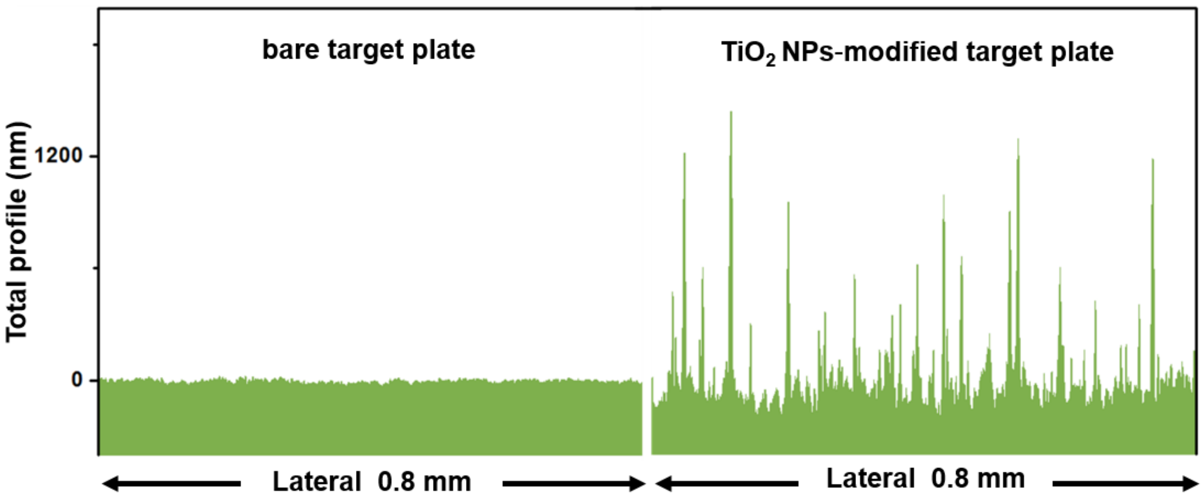

Figure S2 Surface roughness profile of (**a**) a classic bare stainless steel target plate (MSP 96 ground steel MALDI target, Bruker Daltonics), (**b**) a TiO_2 NPs-modified stainless steel target plate.

S3. Comparison of water contact angle on a classic bare and a TiO_2-modified MALDI target plates

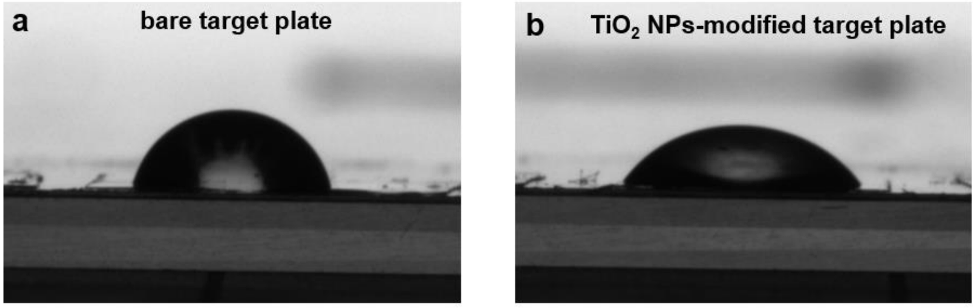

Figure S3 Contact angle of water on the surface of (**a**) a classic bare stainless steel target plate (value 70°), (**b**) a TiO_2 NPs-modified stainless steel target plate (value 38°).

S4. UV-visible absorption spectrum of TiO_2 NPs

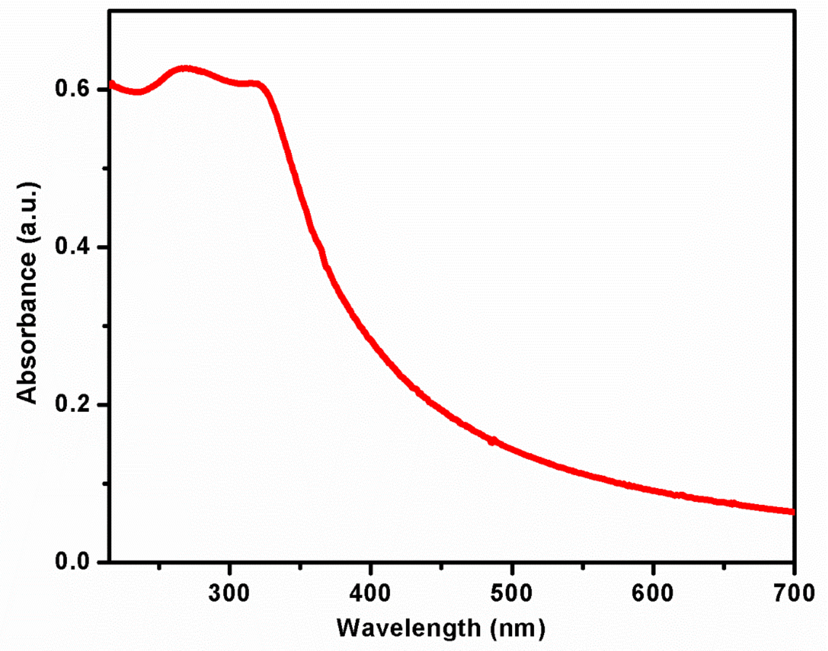

Figure S4 UV-visible absorption spectrum of TiO_2 NPs aqueous suspension (10 μg·mL^{-1}).

TiO_2 has a unique electronic structure, which is characterized by a filled valence band and an empty conduction band. With the band gap of 3.0-3.2 eV (3.0 eV for rutile and 3.2 eV for anatase), TiO_2 have strong absorption in UV range (< 400 nm). MALDI techniques typically use UV lasers such as nitrogen lasers (337.1 nm) and frequency- tripled and quadrupled Nd: YAG lasers (355 nm and 266 nm, respectively). Thus, TiO_2 NPs on the surface of MALDI target plate can absorb energy from the laser source during MALDI-TOF MS measurement.

S5. TiO_2-triggered photocatalytic reactions related to the generation of reactive oxygen species[6]

$$\mathrm{TiO_2} + h\nu \rightarrow \mathrm{e^-} + \mathrm{h^+} \tag{1}$$

$$\mathrm{H_2O\ (surface\ absorbed)} + \mathrm{h^+} \rightarrow {}^{\bullet}\mathrm{OH} + \mathrm{H^+} \tag{2}$$

$$\mathrm{O_2\ (surface\ absorbed)} + \mathrm{e^-} \rightarrow \mathrm{O_2^{\bullet-}} \tag{3}$$

$$\mathrm{O_2^{\bullet-}} + \mathrm{H^+} \rightarrow \mathrm{HO_2^{\bullet}} \tag{4}$$

$$\mathrm{HO_2^{\bullet}} + \mathrm{e^-} + \mathrm{H^+} \rightarrow \mathrm{H_2O_2} \tag{5}$$

S6. Influence of reactive oxygen species scavengers on bacterial fingerprinting patterns

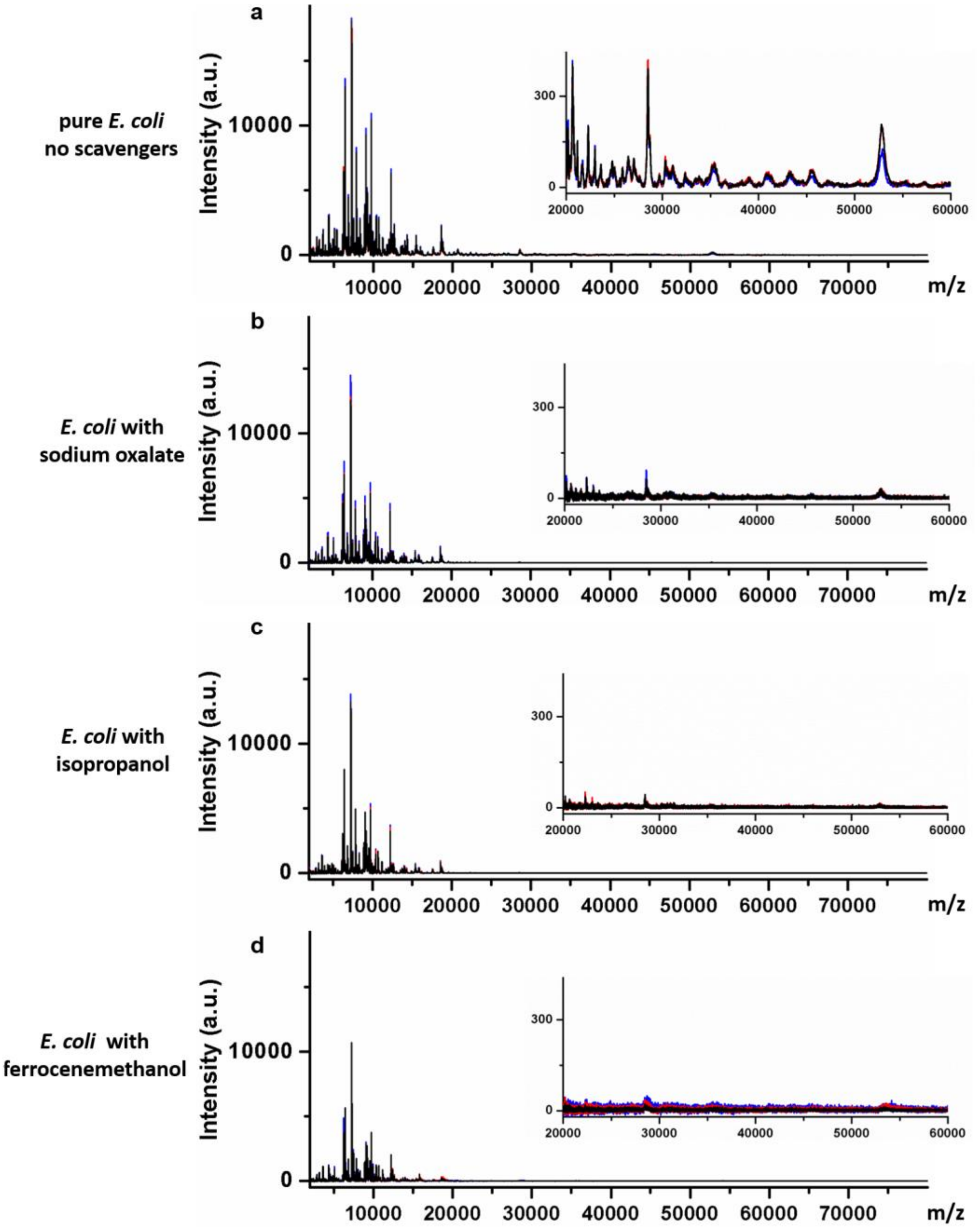

Figure S6 MALDI-TOF MS patterns of intact *Escherichia coli* (*E. coli*, strain DH5α) with the presence of different reactive oxygen species scavengers: (**a**) no scavenger, pure bacteria; (**b**) sodium oxalate (2 mM); (**c**) isopropanol (2 mM); (**d**) ferrocenemethanol (0.2 mM). All of the MALDI-TOF MS measurements were conducted with TiO_2-modified target plates, and were carried out under the exact same instrumental parameters. Number of bacterial cells on each spot was around 5×10^5. Each pattern was the overlay of three replicates (in red, blue and black color, respectively).

S7. MALDI-TOF MS patterns of bacterial protein extracts

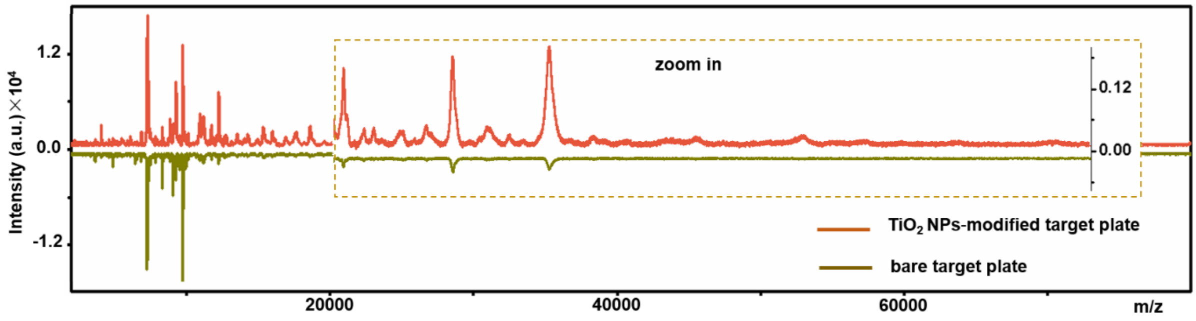

Figure S7 Comparison of MALDI-TOF MS patterns (each was averaged from three replicates) of protein extracts from *Escherichia coli* (strain DH5α) in the mass range of 2,000-80,000 *m/z* obtained by using a classic bare (bottom panel) and a TiO_2 NPs-modified (up panel) target plates. On each sample spot, there is 1 μL of protein extracts and 1 μL of sinapinic acid matrix.

S8. Comparison of bare and TiO_2-modified target plates for the detection of standard protein mixture samples by MALDI-TOF MS

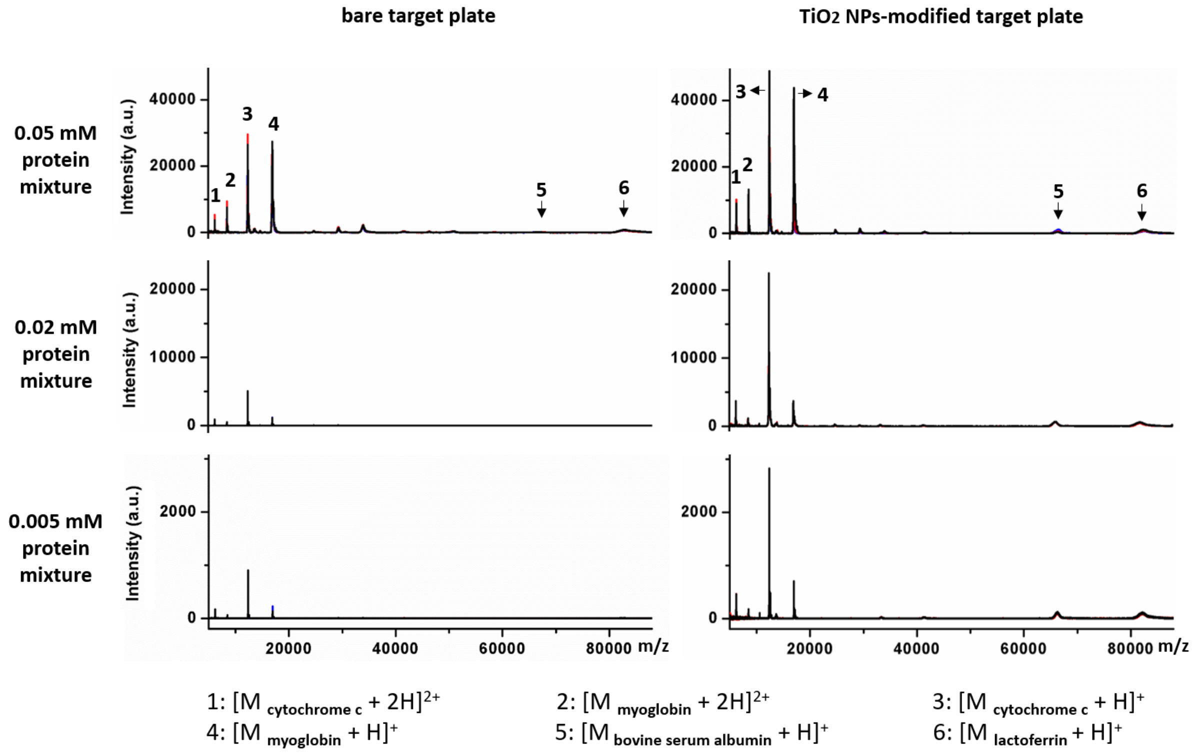

Figure S8 Detection of standard protein mixture samples by MALDI-TOF MS using a bare and a TiO_2 NPs-modified stainless steel target plates. The protein samples contained four different proteins, *i.e.* cytochrome c (~12 kDa), myoglobin (~17 kDa), bovine serum albumin (BSA, ~66 kDa) and lactoferrin (~82 kDa), and were analyzed at three different concentration, *i.e.* 0.05 mM, 0.02 mM and 0.005 mM for each protein. All of the MALDI-TOF MS measurements were conducted with the exactly same instrumental parameters. On each sample spot, there were 1 µL of protein mixture and 1 µL of sinapinic acid matrix. Each pattern was the overlay of three replicates (in red, blue and black color, respectively).

S9. MALDI-TOF MS patterns of intact bacteria using target plate modified with different nanomaterials

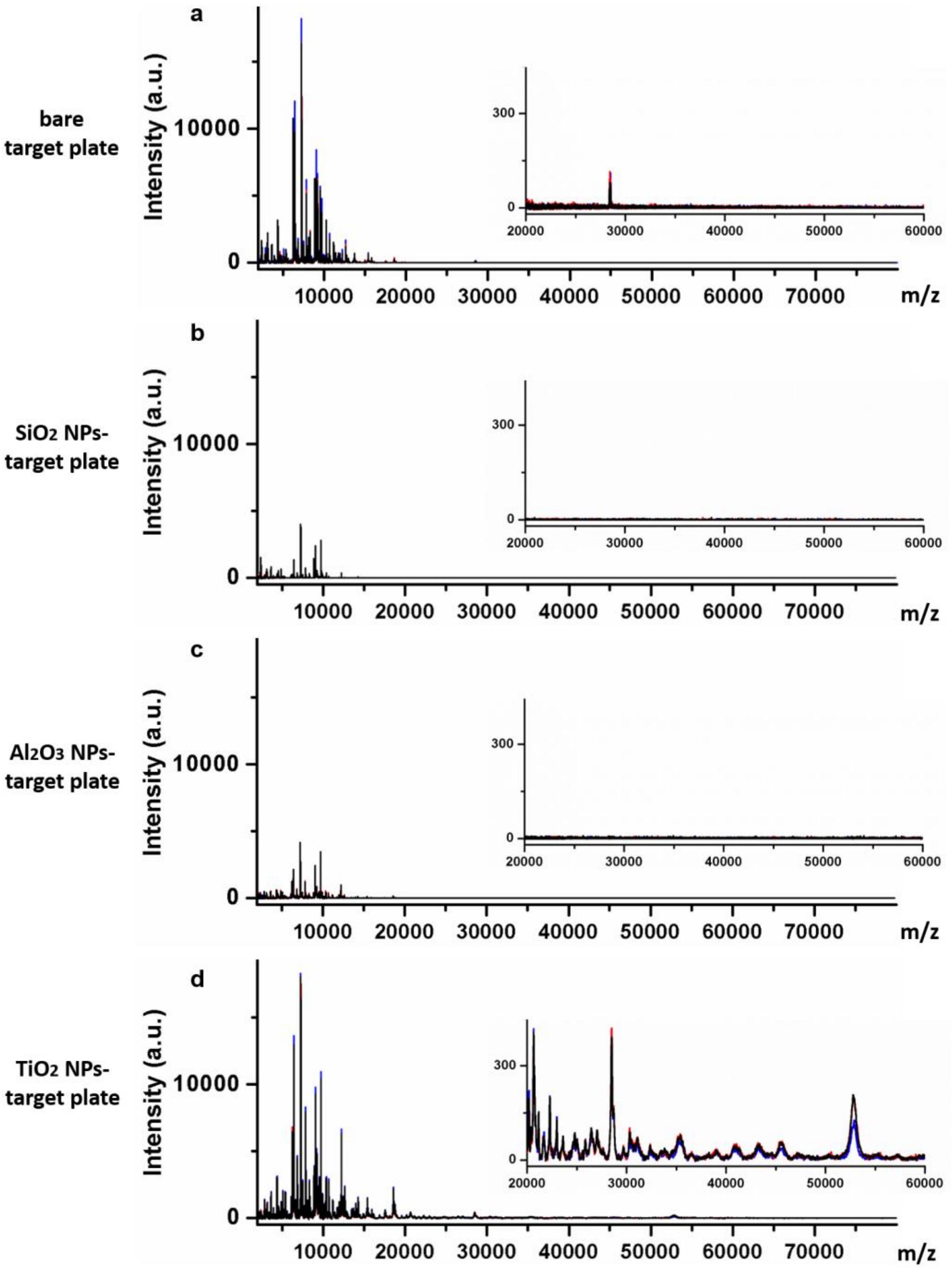

Figure S9 MALDI-TOF MS patterns of intact *Escherichia coli* (strain DH5α) using target plate modified with different nanomaterials: (**a**) bare stainless steel target plate, (**b**) SiO_2 NPs (mesoporous structure, 200 nm particle size, Sigma-Aldrich), (**c**) Al_2O_3 NPs (< 50 nm primary particle size, Sigma-Aldrich), (**d**) TiO_2 NPs (P25, 21 nm primary particle size, Sigma-Aldrich). The SiO_2 and Al_2O_3-modified target plates were prepared in the same way as TiO_2-modified ones. All of the *E. coli* samples and MALDI-TOF MS measurement parameters were exactly the same. Number of bacterial cells on each spot was around 5×10^5. Each pattern was the overlay of three replicates (in red, blue and black color, respectively).

S10. MALDI-TOF MS spectra of standard protein mixtures using target plate modified with different nanomaterials

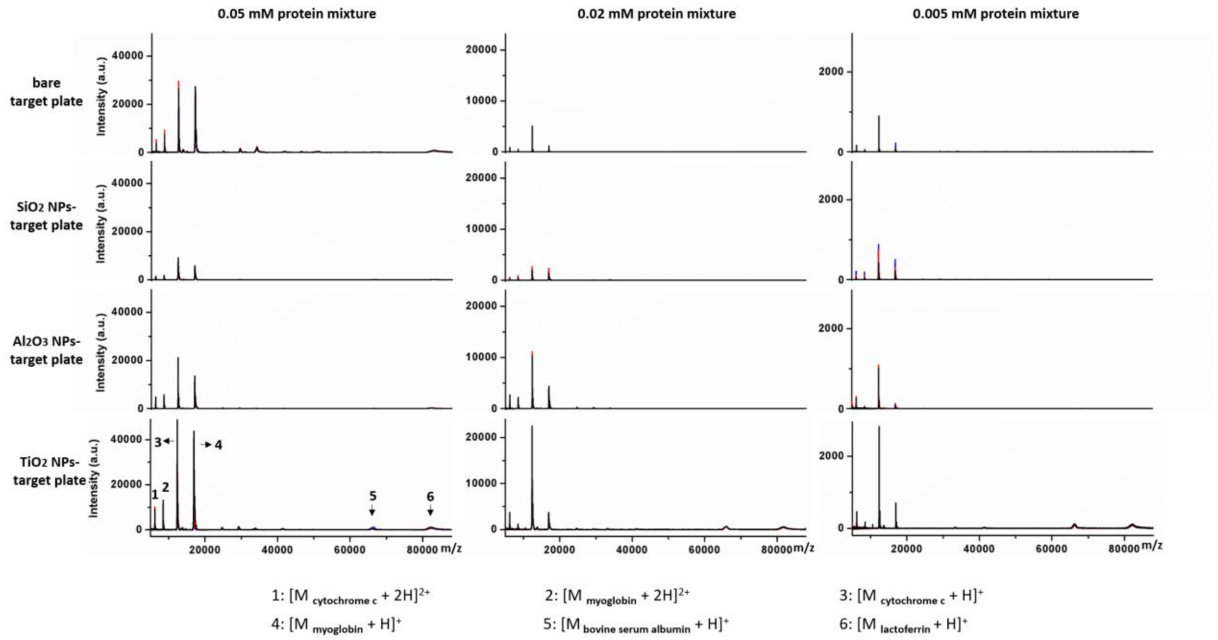

Figure S10 MALDI-TOF MS spectra of standard protein mixtures using target plate modified with different nanomaterials: bare stainless steel target plate, SiO_2 NPs (mesoporous structure, 200 nm particle size, Sigma-Aldrich), Al_2O_3 NPs (< 50 nm primary particle size, Sigma-Aldrich) and TiO_2 NPs (P25, 21 nm primary particle size, Sigma-Aldrich). The SiO_2 and Al_2O_3-modified target plates were prepared in the same way as TiO_2-modified ones. The protein samples contained four different proteins, *i.e.* cytochrome c (~12 kDa), myoglobin (~17 kDa), bovine serum albumin (BSA, ~66 kDa) and lactoferrin (~82 kDa), and were analyzed at three different concentration, *i.e.* 0.05 mM, 0.02 mM and 0.005 mM for each protein. All of the MALDI-TOF MS measurements were conducted with the exactly same instrumental parameters. On each sample spot, there were 1 µL of protein mixture and 1 µL of sinapinic acid matrix. Each pattern was the overlay of three replicates (in red, blue and black color, respectively).

S11. An example of bacterial MALDI-TOF MS fingerprint patterns from three replicates and the averaged pattern

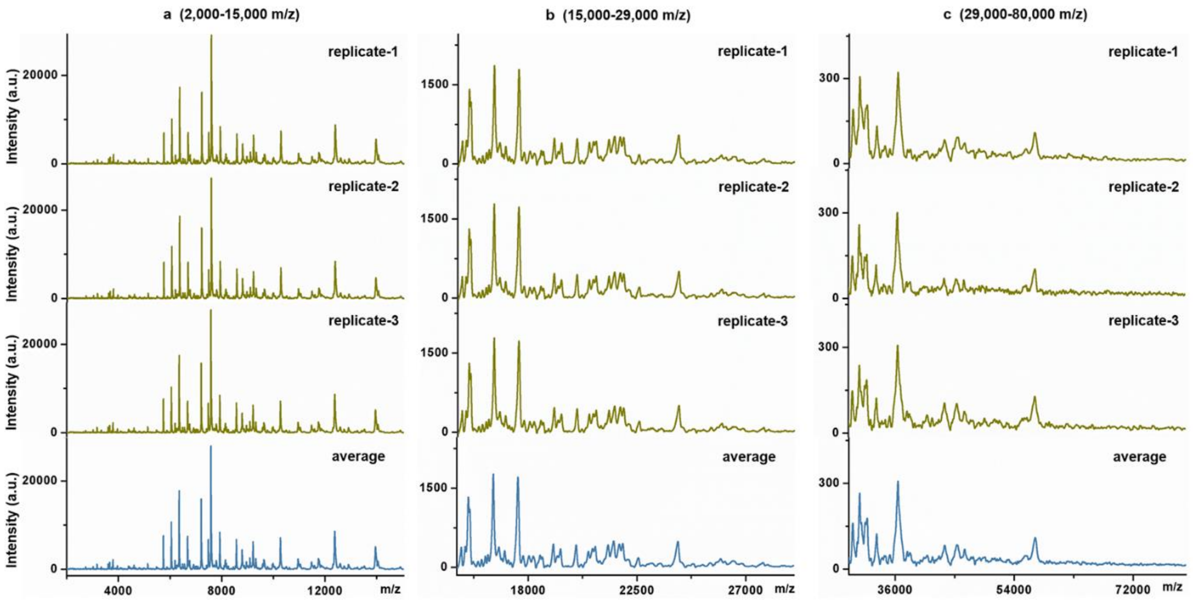

Figure S11 MALDI-TOF MS fingerprint patterns of *Pseudomonas aeruginosa* (strain ATCC 27853) from three replicates and the averaged one. The patterns were shown in three sections: (**a**) 2,000-15,000 *m/z*, (**b**) 15,000-29,000 *m/z*, (**c**) 29,000-80,000 *m/z*. Data were obtained by intact bacteria MALDI-TOF MS fingerprinting using a TiO_2 NPs-modified target plate. Number of bacterial cells on each sample spot was around 5×10^5.

In the present work, each MALDI-TOF MS test was repeated three times; in each replicate, a freshly cultured bacteria strain was measured. Collected bacterial fingerprint patterns demonstrated high reproducibility. For example, in **Fig.S11**, the pattern similarity score is 0.9967 between replicate 1 and 2, 0.9978 between replicate 1 and 3, and 0.9989 between replicate 2 and 3. The similarity scores were calculated using the BacteriaMS platform with cosine correlation algorithm.

S12. A list of proteome database search result

Herein, we show the detailed proteome database search result of the 36 fingerprint peaks newly detected from *Bacillus subtilis* (strain 168) using a TiO_2 NPs-modified target plate in comparison with a classic bare one (in **main article Fig. 3c**). The search was conducted against *Bacillus subtilis* 168 complete proteome database (proteome ID UP000001570) on UniProtKB/Swiss-Prot/TrEMBL database platform. The mass window was selected as 300 ppm (± $m/z_{\text{experimental}}$ × 0.03%) for the mass range of 2,000-80,000 *m/z*.

***m/z* [M+H]⁺ (expriment)**	**MW[1] (Da) (match)**	**Entry[2]**	**Subcellular location**	***pI*[3]**	**GRAVY[4]**
3988±3	3990	MLDVB	information not provided	9.69	-1.211
4375±2	4377	C0H410	information not provided	3.89	-0.267
4774±2	4775	L8EBJ9	cytosol	10.29	-0.512
6157±1	6155	Q7WY62	cytosol	5.68	-0.271
7107±3	7109	P68731	information not provided	5.33	-0.487
7434±3	7431	O31467	cytosol	8.07	0.22
7547±2	7546	O31944	information not provided	5.21	-0.631
8838±3	8836	C0H3T1	information not provided	8.93	0.58
9062±3	9062	O31738	cytosol	6.90	-0.252
9464±2	9462	P96700	information not provided	3.72	-0.527
9670±4	9671	O31846	cytoplasmic membrane	5.15	-0.66
10468±4	10471	P45863	cytoplasmic membrane	9.15	-0.217
10652±3	10652	P32730	information not provided	7.77	-0.187
10959±3	10956	P42924	cytosol	9.84	-0.667
11277±2	11275	P26908	cytosol	9.73	-0.538
12632±3	12635	O32142	periplasmic space	5.72	-0.084
12840±3	12839	O07909	information not provided	9.39	0.052
13475±3	13477	O32184	information not provided	8.88	0.331
14230±2	14227	P36946	cytosol	5.15	0.12
14755±3	14752	O32096	information not provided	5.75	-0.306
14935±3	14931	Q06796	cytosol	9.3	-0.06
15081±4	15077	O31914	information not provided	4.44	-0.496
17781±4	17782	O31705	cytosol	4.94	-0.517
18092±3	18091	O32230	cytosol	9.99	-0.888
18246±4	18250	O07515	cytoplasmic membrane	10.73	1.28
19405±4	19407	O07582	cytoplasmic membrane	6.65	-0.448
19917±3	19921	P96654	information not provided	5.71	-0.047
20163±4	20160	P54516	cytoplasmic membrane	5.91	0.518
21716±3	21716	O32216	cytoplasmic membrane	8.93	0.991
22346±4	22340	P81102	cytosol	5.41	-0.219

22931±5	22929	P25053	cytosol	7.89	-0.181
22962±4	22966	P50741	cytoplasmic membrane	9.21	0.775
24247±4	24245	P55184	cytosol	5.49	-0.153
15960±3	un-assigned, no possible match				
16325±3	un-assigned, more than one possible match (MW:16324 Da, MW: 16325 Da)				
16655±4	un-assigned, more than one possible match (MW:16650 Da, MW:16659 Da)				

MW[1]: molecular weight
Entry[2]: unique and stable entry identifier (also known as accession number) of a protein in UniProtKB/Swiss-Prot /TrEMBL database
p*I*[3]: isoelectric point
GRAVY[4]: grand average value of hydropathicity

S13. Characterization of the newly detected MS peaks from *B. subtilis* 168 with TiO_2-modified target plate by proteome database search

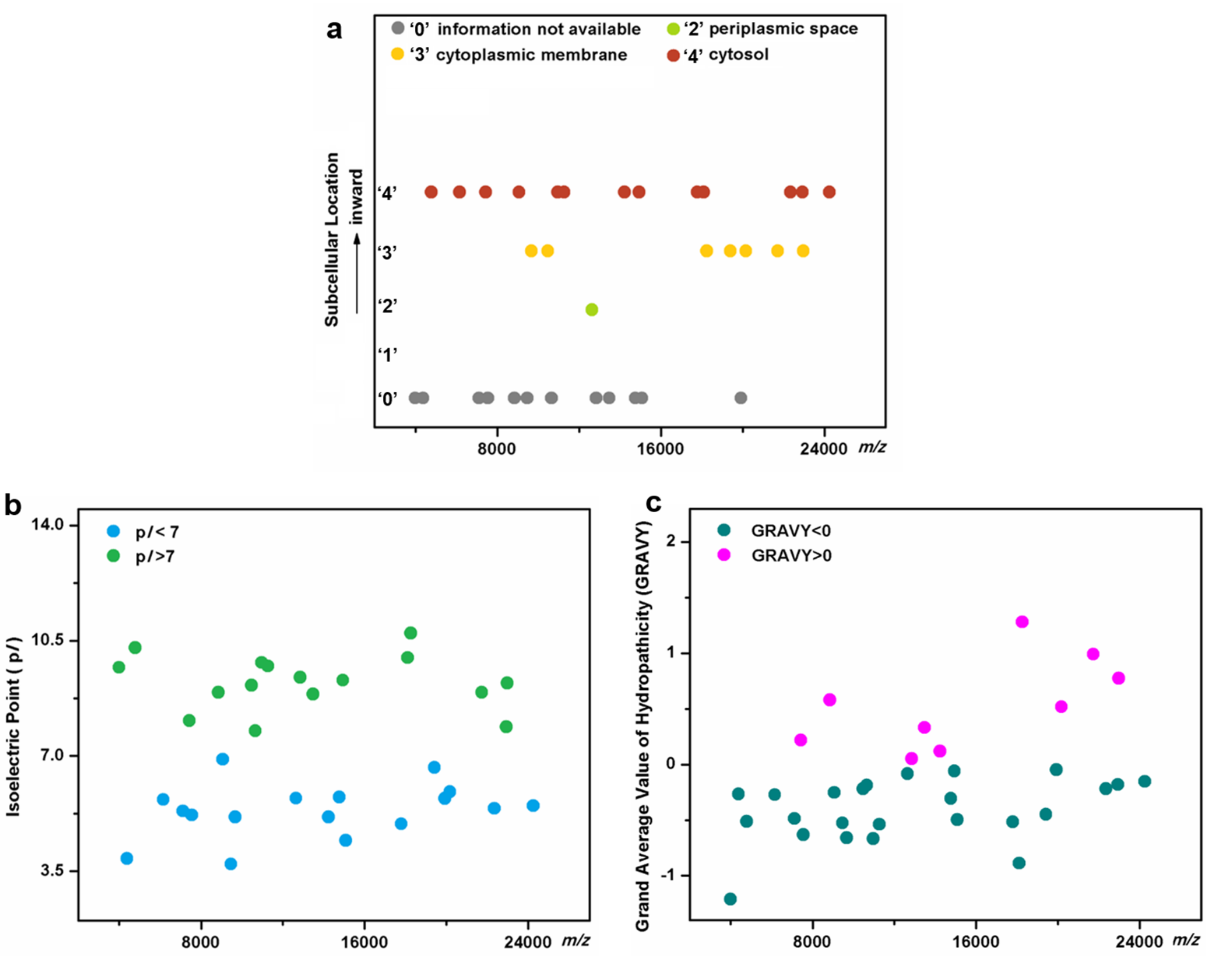

Figure S13 The 33 tentatively assigned proteins, newly detected from *B. subtilis* using the TiO_2 NPs-modified target plate in comparison with a classic bare one, were characterized by their (**a**) subcellular location ('0': related information was not provided in the database, '1': the outer peptidoglycan layer, '2': the periplasmic space between peptidoglycan layer and cytoplasmic membrane, '3': cytoplasmic membrane, '4': interior region, cytosol), (**b**) isoelectric point (p*I*) and (**c**) grand average value of hydropathicity (GRAVY).

S14. Unsuccessful detection of antimicrobial resistance-associated proteins by using classic bare stainless steel target plates

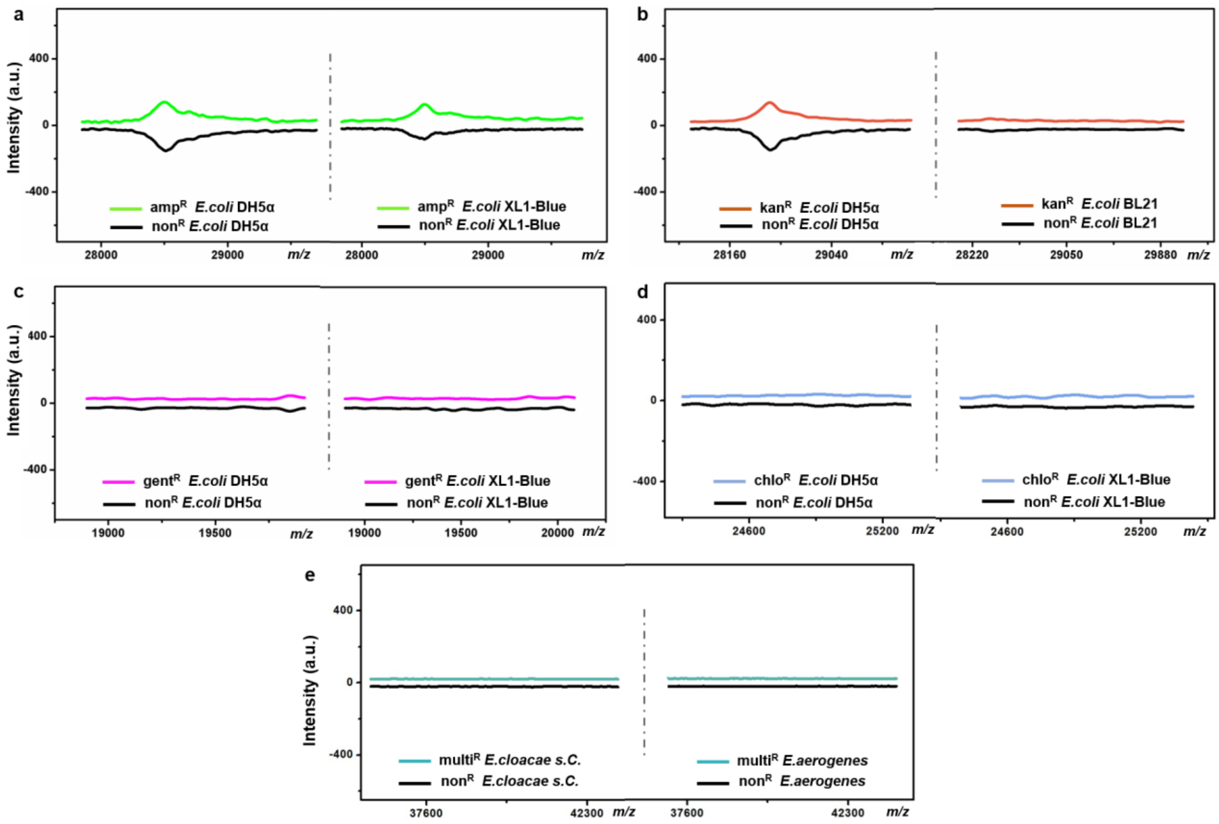

Figure S14 Classic bare stainless steel plates were used to detect antimicrobial resistance-associated proteins from (**a**) ampicillin-resistant (amp^R), (**b**) kanamycin-resistant (kan^R), (**c**) gentamicin-resistant (gent^R), (**d**) chloramphenicol-resistant (chlo^R) *E. coli* strains (two strains in each case) and (**e**) multidrug-resistant (multi^R) *E. cloacae s. C.* and *E. aerogenes* by intact bacteria MALDI-TOF MS. Each pattern was averaged from three replicates. Number of bacterial cells on each sample spot was around 5×10^5.

In the main article, it has been demonstrated that five different types of antimicrobial resistance-associated proteins were successfully detected from intact bacteria cells by MADLI-TOF MS fingerprinting method when TiO_2-modified target plates were used, as shown in Fig.4. As a comparison, all the measurements in Fig.4 were repeated with classic bare stainless steel plates. The mass spectra in corresponding mass range were shown in Figure S14. Clearly, none of the protein markers were detected with classic bare stainless steel plates, confirming the advantages of TiO_2-modifed target plates.

S15. LC-MS/MS analysis of SDS-PAGE gel bands

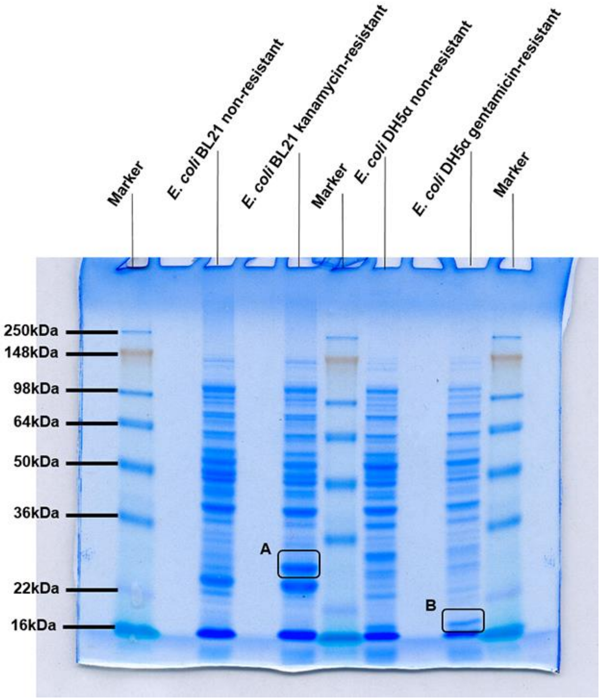

Figure S15 Coomassie blue stained sodium dodecyl sulfate polyacrylamide gel electrophoresis gel of kanamycin-resistant *E.coli* BL21, non-resistant *E.coli* BL21, gentamicin-resistant *E.coli* DH5α and non-resistant *E.coli* DH5α. A ~29 kDa band (A) and a ~19 kDa band (B) were clearly observed on the kanamycin-resistant *E.coli* BL21 lane and the gentamicin-resistant *E.coli* DH5α lane, respectively.

Table S1 Gentamicin acetyltransferase I peptides detected from the ~19 kDa band of gentamicin-resistant *E.coli* DH5α by in-gel digest and LC-MS/MS.

Gentamicin acetyltransferase I; UniProt access P23181; 100% protein identification probability; 19,442.7 Da;

60 exclusive unique peptides; 114 exclusive unique spectrum; 295 total spectra; 164/177 amino acids (93% coverage);

Protein sequence (covered sequence is highlighted in red):

MLRSSNDVTQ QGSRPKTKLG GSSMGIIRTC RLGPDQVKSM RAALDLFGRE FGDVATYSQH QPDSDYLGNL LRSKTFIALA AFDQEAVVGA LAAYVLPRFE QPRSEIYIYD LAVSGEHRRQ GIATALINLL KHEANALGAY VIYVQADYGD DPAVALYTKL GIREEVMHFD IDPSTAT

Observed peptides		Observed m/z value			
		$[M+2H]^{2+}$	$[M+3H]^{3+}$	$[M+4H]^{4+}$	$[M+5H]^{5+}$
(R)SSNDVTQQGSR(P)		589.77	393.52		
(R)SSNDVTQQGSRPK(T)		702.35	468.57	351.68	
(R)sSNDVTQQGSRPK(T)	Gln->pyro-Glu(-17)		462.89		
(R)sSNDVTQQGSRPK(T)	Ammonia-loss (-17)		463.23		
(S)SNDVTQQGSRPK(T)			439.56		
(S)NDVTQQGSRPK(T)			410.55		
(K)TKLGGSSMGIIR(T)			407.23		
(K)LGGSSMGIIR(T)		495.77	330.85		
(K)LGGSSmGIIR(T)	Oxidation (+16)	503.77	336.18		
(R)LGPDQVK(S)		378.72			
(R)AALDLFGR(E)		431.74			
(R)AALDLFGREFGDVATYSQHQPDSDYLGNLLR(S)				868.43	
(R)EFGDVATYSQHQPD(S)		797.35			
(R)eFGDVATYSQHQPDSDYLGN(L)	Dehydrated (-18)	1,112.98	742.32		
(R)EFGDVATYSQHQPDSDYLGN(L)		1,121.98			
(R)EFGDVATYSQHQPDSDYLGNLLR(S)		1,313.12	875.75	657.06	525.85
(R)eFGDVAtYSQHQPDSDYLGNLLR(S)	Ammonia-loss (-17), Phospho (+80)		897.05		
(R)eFGDVATYSQHQPDSDYLGNLLR(S)	Gln->pyro-Glu (-17)		870.08		
(R)EFGDVAtYSQHQPDSDYLGNLLR(S)	Phospho (+80)		903.07	677.31	
(R)eFGDVATYSQHQPDSDYLGNLLR(S)	Dehydrated (-18)	1,304.12		652.56	
(E)FGDVATYSQHQPDSDYLGNLLR(S)			832.73		
(A)TYSQHQPDSDYLGNLLR(S)			669.66		
(T)YSQHQPDSDYLGNLLR(S)		953.46			
(Y)SQHQPDSDYLGNLLR(S)		871.93			

(S)QHQPDSDYLGNLLR(S)			552.61		
(H)qPDSDYLGNLLR(S)	Ammonia-loss (-17)	687.34			
(H)QPDSDYLGNLLR(S)		695.85	464.24		
(D)SDYLGNLLR(S)		525.78			
(K)TFIALAAFDQEAVVGA(L)		811.93			
(K)TFIALAAFDQEAVVGAL(A)			579.31		
(K)TFIALAAFDQEAVVGALAAY(V)		1,021.04			
(L)AAYVLPRFEQPRSEIYIYDLAVSGEHR(R)				796.16	
(P)RSEIYIYDLAVSGEHR(R)			636.66	477.75	
(R)SEIYIYDLAVSGEH(R)		798.38	532.59		
(R)SEIYIYDLAVSGEHR(R)		876.43	584.63	438.72	
(R)sEIYIYDLAVSGEHR(R)	Phospho (+80)		611.62		
(Y)IYDLAVSGEHR(R)		630.32	420.55		
(Y)DLAVSGEHR(R)			328.50		
(D)LAVSGEHR(R)		434.74			
(R)RQGIATAL(I)		415.25			
(R)RQGIATALINLLK(H)		706.45	470.97		
(R)rQGIATALINLLK(H)	Gln->pyro-Glu (-17)	697.43	465.29		
(R)QGIATALIN(L)		450.76			
(R)qGIATALINLLK(H)	Gln->pyro-Glu (-17)	619.38	413.26		
(R)QGIATALINLLK(H)		627.89	418.93		
(Q)GIATALINLLK(H)		563.86	376.24		
(G)IATALINLLK(H)		535.35			
(I)ATALINLLK(H)		478.81			
(A)TALINLLK(H)		443.29			
(K)HEANALGAY(V)		473.23			
(K)HEANALGAYVIY(V)		660.83	440.89		
(K)HEANALGAYVIYV(Q)		710.37			
(K)HEANALGAYVIYVQ(A)		774.40			
(K)HEANALGAYVIYVQADYGD(D)		1,034.99			
(K)HEANALGAYVIYVQADYGDD(P)		1,092.50	728.67		
(K)HEANALGAYVIYVQADYGDDPAVA(L)			841.74		
(K)HEANALGAYVIYVQADYGDDPAVAL(Y)		1,318.14	879.43		
(K)HEANALGAYVIYVQADYGDDPAVALY(T)			933.45	700.34	
(K)HEANALGAYVIYVQADYGDDPAVALYT(K)			967.13		
(K)HEANALGAYVIYVQADYGDDPAVALYTK(L)		1,515.25	1,009.83	757.62	606.30
(K)hEANALGAyVIYVQADYGDDPAVALYTK(L) Gln->pyro-Glu (-17), Phospho (+80)			1,030.81		
(K)hEANALGAYVIYVQADYGDDPAVALYTK(L) Gln->pyro-Glu (-17)			1,004.16		
(K)HEANALGAYVIYVQADYGDDPAVALYTk(L)	Acetyl (+42)			768.63	
(K)HEANALGAyVIYVQADYGDDPAVALYTK(L)	Phospho (+80)		1,037.15	778.12	
(K)HEANALGAYVIYVQADYGDDPAVALYTKLG(I)			1,066.87		

(E)ANALGAYVIYVQADYGDDPAVALYTK(L)		921.47		
(N)ALGAYVIYVQADYGDDPAVALYTK(L)	1,288.66	859.44	644.83	
(L)GAYVIYVQADYGDDPAVALYTK(L)	1,196.59			
(A)YVIYVQADYGDDPAVALYTK(L)	1,132.56	755.38		
(Y)VIYVQADYGDDPAVALYTK(L)	1,051.03	701.02	526.02	
(Y)VQADYGDDPAVALYTK(L)	863.42	575.95		
(G)DDPAVALYTK(L)	546.78			
(D)PAVALYTK(L)	431.76			
(R)EEVMHFDIDPSTA(-)	745.83			
(R)EEVMHFDIDPSTAT(-)	796.35			
(R)EEVmHFDIDPSTAT(-) Oxidation (+16)	804.35	536.57		
(M)HFDIDPSTAT(-)	552.26			

Table S2 Neomycin-kanamycin phosphotransferase type II peptides detected from the ~29 kDa band of kanamycin-resistant *E.coli* BL21 by in-gel digest and LC-MS/MS.

Neomycin-kanamycin phosphotransferase type II; UniProt access P00552; 100% protein identification probability; 29,048.4 Da; 72 exclusive unique peptides; 116 exclusive unique spectrum; 206 total spectra; 246/264 amino acids (93% coverage);

Protein sequence (covered sequence is highlighted in red):

MIEQDGLHAG SPAAWVERLF GYDWAQQTIG CSDAAVFRLS AQGRPVLFVK TDLSGALNEL QDEAARLSWL

ATTGVPCAAV LDVVTEAGRD WLLLGEVPGQ DLLSSHLAPA EKVSIMADAM RRLHTLDPAT CPFDHQAKHR

IERARTRMEA GLVDQDDLDE EHQGLAPAEL FARLKARMPD GEDLVVTHGD ACLPNIMVEN GRFSGFIDCG

RLGVADRYQD IALATRDIAE ELGGEWADRF LVLYGIAAPD SQRIAFYRLL DEFF

Observed peptides	Observed *m/z* value			
	$[M+2H]^{2+}$	$[M+3H]^{3+}$	$[M+4H]^{4+}$	$[M+5H]^{5+}$
(-)MIEQDGLHAGSPAAWVER(L)	983.98	656.32		
(-)mIEQDGLHAGSPAAWVER(L) Oxidation (+16)	991.97	661.65		
(-)mIEQDGLHAGSPAAWVER(L) Acetyl (+42)	1,005.48	670.66		
(-)MIEQDGLHAGsPAAWVER(L) Phospho (+80)		683.31		
(M)IEQDGLHAGSPAAWVER(L)	918.46	612.64		
(Q)DGLHAGSPAAWVER(L)	733.37	489.24		
(D)GLHAGSPAAWVER(L)	675.85	450.90		

(G)LHAGSPAAWVER(L)		431.89		
(L)HAGSPAAWVER(L)	590.80	394.20		
(H)AGSPAAWVER(L)	522.27			
(G)SPAAWVER(L)	458.24			
(R)LFGYDWAQQTIGcSDAA(V) Carbamidomethyl (+57)	951.92			
(R)LFGYDWAQQTIGcSDAAVFR(L) Carbamidomethyl (+57)	1,153.04	769.03	577.02	
(R)lFGyDWAQQTIGcSDAAVFR(L)<br>Ammonia-loss (-17), Phospho (+80), Carbamidomethyl (+57)	1,185.00			
(F)GYDWAQQTIGcSDAAVFR(L) Carbamidomethyl (+57)	1,022.97	682.31		
(G)YDWAQQTIGcSDAAVFR(L) Carbamidomethyl (+57)	994.45			
(W)AQQTIGcSDAAVFR(L) Carbamidomethyl (+57)	762.37			
(Q)TIGcSDAAVFR(L) Carbamidomethyl (+57)	598.79			
(I)GcSDAAVFR(L) Carbamidomethyl (+57)	491.73			
(R)LSAQGRPVLFVK(T)		438.94		
(R)PVLFVKTDLSGALNELQDEAAR(L)		796.10		
(K)TDLSGALNELQDEAAR(L)	851.92	568.28		
(D)LSGALNELQDEAAR(L)	743.88			
(L)SGALNELQDEAAR(L)	687.34			
(R)LSWLATTGVPcAAVLDVVTEAGR(D)<br>Carbamidomethyl (+57)	1,193.63	796.09	597.32	
(R)lsWLAtTGVPcAAVLDVVTEAGR(D)<br>Ammonia-loss (-17), Phospho (+80), Phospho (+80),<br>Carbamidomethyl (+57)		843.73		
(R)lsWLATTGVPcAAVLDVVTEAGR(D)<br>Ammonia-loss (-17), Phospho (+80), Carbamidomethyl (+57)		817.40		
(S)WLATTGVPcAAVLDVVTEAGR(D) Carbamidomethyl (+57)	1,094.07	729.38		
(W)LATTGVPcAAVLDVVTEAGR(D) Carbamidomethyl (+57)	1,000.53	667.36		
(L)ATTGVPcAAVLDVVTEAGR(D) Carbamidomethyl (+57)	943.99	629.66		
(A)TTGVPcAAVLDVVTEAGR(D) Carbamidomethyl (+57)		605.98		
(T)TGVPcAAVLDVVTEAGR(D) Carbamidomethyl (+57)	857.95	572.30		
(T)GVPcAAVLDVVTEAGR(D) Carbamidomethyl (+57)	807.42	538.62		
(G)VPcAAVLDVVTEAGR(D) Carbamidomethyl (+57)	778.91	519.61		
(C)AAVLDVVTEAGR(D)	600.83	401.23		
(C)AAVLDVVTEAGRDWLLLGEVPGQDLLSSHLAPAEK(V)			918.25	
(A)VLDVVTEAGR(D)	529.80			
(R)DWLLLGEVPGQDLL(S)	784.42			
(R)DWLLLGEVPGQDLLSSH(L)	939.99	626.99		
(R)DWLLLGEVPGQDLLSSHLAPAEK(V)	1,244.66	830.11	622.83	
(R)dWLLLGEVPGQDLLSSHLAPAEK(V) Ammonia-loss (-17)		825.10		
(R)DWLLLGEVPGQDLLSsHLAPAEK(V) Phospho (+80)		857.11		
(D)WLLLGEVPGQDLLSSHLAPAEK(V)		791.77		
(W)LLLGEVPGQDLLSSHLAPAEK(V)		729.74		
(L)LLGEVPGQDLLSSHLAPAEK(V)	1,037.57	692.05		

(L)LGEVPGQDLLSSHLAPAEK(V)		654.35		
(L)GEVPGQDLLSSHLAPAEK(V)	924.98			
(G)EVPGQDLLSSHLAPAEK(V)		597.65		
(V)PGQDLLSSHLAPAEK(V)	781.92			
(Q)DLLSSHLAPAEK(V)		427.57		
(D)LLSSHLAPAEK(V)	583.33			
(L)SSHLAPAEK(V)	470.25	313.84		
(S)HLAPAEK(V)	383.22			
(K)VSIMADAMR(R)	497.25			
(K)VSIMADAmR(R) Oxidation (+16)	505.25			
(K)VSImADAmR(R) Oxidation (+16), Oxidation (+16)	513.24	342.50		
(R)RLHTLDPATcPFDHQAK(H) Carbamidomethyl (+57)			502.50	
(R)LHTLDPAT(C)	434.23			
(R)LHTLDPATcPFDH(Q) Carbamidomethyl (+57)		508.57		
(R)LHTLDPATcPFDHQAK(H) Carbamidomethyl (+57)	925.95	617.63	463.48	370.98
(R)LHTLDPATcPFDHQAKHR(I) Carbamidomethyl (+57)				429.62
(H)TLDPATcPFDHQAK(H) Carbamidomethyl (+57)	800.88	534.25		
(D)PATcPFDHQAK(H) Carbamidomethyl (+57)		424.53		
(P)ATcPFDHQAK(H) Carbamidomethyl (+57)	587.77			
(A)TcPFDHQAK(H) Carbamidomethyl (+57)	552.25			
(R)MEAGLVDQDDLDEEHQGLAPAELFAR(L)		957.18	718.09	
(R)mEAGLVDQDDLDEEHQGLAPAELFAR(L) Oxidation (+16)		962.45	722.33	
(D)DLDEEHQGLAPAELFAR(L)		637.98		
(D)LDEEHQGLAPAELFAR(L)		599.30		
(E)eHQGLAPAELFAR(L) Dehydrated (-18)	710.87			
(Q)GLAPAELFAR(L)	522.80			
(M)PDGEDLVVTHGDAcLPNIMVENGR(F) Carbamidomethyl (+57)		870.08		
(C)LPNIMVENGR(F)	571.80			
(R)FSGFIDcGR(L) Carbamidomethyl (+57)	529.74			
(F)SGFIDcGR(L) Carbamidomethyl (+57)	456.21			
(R)YQDIALATR(D)	525.78	350.86		
(R)DIAEELGGEWADR(F)	730.84	487.56		
(R)DIAEELGGEWADRFLVLYGIAAPDSQR(I)		998.50		
(R)FLVLYGIAAPDSQR(I)	775.42	517.28		
(F)LVLYGIAAPDSQR(I)	701.89			
(L)VLYGIAAPDSQR(I)	645.34			
(Y)GIAAPDSQR(I)	457.74			
(R)IAFYRLLDEFF(-)	717.38			

S16. Lists of antimicrobial susceptibility profiles

Results of antimicrobial susceptibility testing conducted with bioMérieux VITEK 2 automated AST system, based on antimicrobial drugs culture method, are listed in **Table S1-S12**.

Table S1 Antimicrobial susceptibility profile of *Enterobacter cloacae ssp. cloacae* before the transfer of gene *AmpC* (test duration 9.00h)

**Antibiotic**	**MIC* μg·mL^{-1}**	**Interpretation***	**Antibiotic**	**MIC* μg·mL^{-1}**	**Interpretation***
Amoxicillin	--	R	Ertapenem	≤ 0.5	S
Ampicillin	16	R	Imipenem	≤ 0.25	S
Amoxicillin/ clavulanic acid	≥ 32	R	Meropenem	--	S
Cefuroxime	--	I	Gentamicin	≤ 1	S
Cefuroxime Axetil	--	I	Ciprofloxacin	≤ 0.25	S
Cefoxitine	--	I	Norfloxacin	≤ 0.5	S
Cefpodoxime	--	I	Tetracycline	≤ 1	S
Ceftazidime	≤ 1	S	Fosfomycin	≤ 16	S
Ceftriaxone	≤ 1	S	Nitrofurantoin	≤ 16	S
Cefepime	≤ 1	S	Trimethoprim/ Sulfamethoxazole	≤ 20	S
Piperacillin/ tazobactam	≤ 4	S			

Table S2 Antimicrobial susceptibility profile of *Enterobacter cloacae ssp. cloacae* after the transfer of gene *AmpC* (test duration 9.50h)

**Antibiotic**	**MIC* μg·mL^{-1}**	**Interpretation***	**Antibiotic**	**MIC* μg·mL^{-1}**	**Interpretation***
Amoxicillin	--	R	Ertapenem	1	I
Ampicillin	≥ 32	R	Imipenem	≤ 0.25	S
Amoxicillin/ clavulanic acid	≥ 32	R	Meropenem	--	S
Cefuroxime	≥ 64	R	Gentamicin	≤ 1	S
Cefuroxime Axetil	≥ 64	R	Ciprofloxacin	≤ 0.25	S
Cefoxitine	≥ 64	R	Norfloxacin	≤ 0.5	S
Cefpodoxime	≥ 8	R	Tetracycline	2	S
Ceftazidime	≥ 64	R	Fosfomycin	≤ 16	S
Ceftriaxone	≥ 64	R	Nitrofurantoin	32	S
Cefepime	2	I	Trimethoprim/ Sulfamethoxazole	≤ 20	S
Piperacillin/ tazobactam	≥ 128	R			

Table S3 Antimicrobial susceptibility profile of *Enterobacter aerogenes* before the transfer of gene *AmpC* (test duration 9.25h)

Antibiotic	MIC* µg·mL^{-1}	Interpretation*	Antibiotic	MIC* µg·mL^{-1}	Interpretation*
Amoxicillin	--	R	Ertapenem	≤ 0.5	S
Ampicillin	8	R	Imipenem	≤ 0.5	S
Amoxicillin/ clavulanic acid	16	R	Meropenem	--	S
Cefuroxime	4	S	Gentamicin	≤ 1	S
Cefuroxime Axetil	4	S	Ciprofloxacin	≤ 0.25	S
Cefoxitine	--	I	Norfloxacin	≤ 0.5	S
Cefpodoxime	≤ 0.05	S	Tetracycline	≤ 1	S
Ceftazidime	≤ 1	S	Fosfomycin	≤ 16	S
Ceftriaxone	≤ 1	S	Nitrofurantoin	≤ 16	S
Cefepime	≤ 1	S	Trimethoprim/ Sulfamethoxazole	≤ 20	S
Piperacillin/ tazobactam	≤ 4	S			

Table S4 Antimicrobial susceptibility profile of *Enterobacter aerogenes* after the transfer of gene *AmpC* (test duration 9.25h)

Antibiotic	MIC* µg·mL^{-1}	Interpretation*	Antibiotic	MIC* µg·mL^{-1}	Interpretation*
Amoxicillin	--	R	Ertapenem	≤ 0.5	S
Ampicillin	≥ 32	R	Imipenem	≤ 0.25	S
Amoxicillin/ clavulanic acid	≥ 32	R	Meropenem	--	S
Cefuroxime	≥ 64	R	Gentamicin	≤ 1	S
Cefuroxime Axetil	≥ 64	R	Ciprofloxacin	≤ 0.25	S
Cefoxitine	≥ 64	R	Norfloxacin	≤ 0.5	S
Cefpodoxime	≥ 8	R	Tetracycline	≤ 1	S
Ceftazidime	≥ 64	R	Fosfomycin	≤ 16	S
Ceftriaxone	16	R	Nitrofurantoin	32	S
Cefepime	2	I	Trimethoprim/ Sulfamethoxazole	≤ 20	S
Piperacillin/ tazobactam	≥ 128	R			

Table S5 Antimicrobial susceptibility profile of *Escherichia coli* ATCC25922 (test duration 10.25h)

Antibiotic	MIC* μg·mL^{-1}	Interpretation*	Antibiotic	MIC* μg·mL^{-1}	Interpretation*
Amoxicillin	--	R	Ertapenem	≤ 0.5	S
Ampicillin	4	S	Imipenem	≤ 0.25	S
Amoxicillin/ clavulanic acid	4	S	Meropenem	--	S
Cefuroxime	4	S	Gentamicin	≤ 1	S
Cefuroxime Axetil	4	S	Ciprofloxacin	≤ 0.25	S
Cefoxitine	≤ 4	S	Norfloxacin	≤ 0.5	S
Cefpodoxime	≤ 0.25	S	Tetracycline	≤ 1	S
Ceftazidime	≤ 1	S	Fosfomycin	≤ 16	S
Ceftriaxone	≤ 1	S	Nitrofurantoin	≤ 16	S
Cefepime	≤ 1	S	Trimethoprim/ Sulfamethoxazole	≤ 20	S
Piperacillin/ tazobactam	≤ 4	S			

Table S6 Antimicrobial susceptibility profile of CTX-M type extended-spectrum β-lactamase-producing *Escherichia coli* (test duration 8.75h)

Antibiotic	MIC* μg·mL^{-1}	Interpretation*	Antibiotic	MIC* μg·mL^{-1}	Interpretation*
Amoxicillin	--	R	Ertapenem	≤ 0.5	S
Ampicillin	≥ 32	R	Imipenem	≤ 0.25	S
Amoxicillin/ clavulanic acid	16	R	Meropenem	--	S
Cefuroxime	≥ 64	R	Gentamicin	≥ 64	R
Cefuroxime Axetil	≥ 64	R	Ciprofloxacin	≥ 4	R
Cefoxitine	32	I	Norfloxacin	≥ 16	R
Cefpodoxime	≥ 8	S	Tetracycline	≥ 16	R
Ceftazidime	16	R	Fosfomycin	≤ 16	S
Ceftriaxone	≥ 64	R	Nitrofurantoin	≤ 16	S
Cefepime	8	R	Trimethoprim/ Sulfamethoxazole	≥ 320	R
Piperacillin/ tazobactam	8	S			

Table S7 Antimicrobial susceptibility profile of *Pseudomonas aeruginosa* ATCC 27853 (test duration 17 h)

**Antibiotic**	**MIC* µg·mL^{-1}**	**Interpretation***	**Antibiotic**	**MIC* µg·mL^{-1}**	**Interpretation***
Ticarcillin	≤ 8	S	Amikacin	≤ 2	S
Ticarcillin/ clavulanic acid	16	S	Gentamicin	≤ 1	S
Piperacillin	≤ 4	S	Netilmicin	--	S
Piperacillin/ tazobactam	≤ 4	S	Tobramycin	≤ 1	S
Ceftazidime	≤ 1	S	Ciprofloxacin	≤ 0.25	S
Cefepime	≤ 1	S	Levofloxacin	≤ 0.12	S
Aztreonam	2	I	Colistin	≤ 0.5	S
Imipenem	1	S	Rifampicin	--	--
Meropenem	≤ 0.25	S	Trimethoprim/ Sulfamethoxazole	≥ 320	R

Table S8 Antimicrobial susceptibility profile of multidrug-resistant *Pseudomonas aeruginosa* (test duration 10.75h)

**Antibiotic**	**MIC* µg·mL^{-1}**	**Interpretation***	**Antibiotic**	**MIC* µg·mL^{-1}**	**Interpretation***
Ticarcillin	--	--	Amikacin	--	R
Ticarcillin/ clavulanic acid	--	--	Gentamicin	--	R
Piperacillin	--	--	Netilmicin	--	R
Tazobacam	--	R	Tobramycin	--	R
Ceftazidime	--	R	Ciprofloxacin	--	--
Cefepime	--	R	Levofloxacin	--	--
Aztreonam	--	--	Colistin	--	S
Imipenem	--	--	Rifampicin	--	--
Meropenem	--	S	Trimethoprim/ Sulfamethoxazole	--	--

Table S9 Antimicrobial susceptibility profile of *Staphylococcus aureus* ATCC 29213 (test duration 9.75h)

**Antibiotic**	**MIC* µg·mL^{-1}**	**Interpretation***	**Antibiotic**	**MIC* µg·mL^{-1}**	**Interpretation***
Test Cefoxitine Screen	--	Negative	Erythromycin	--	S
Benzylpenicillin	0.06	S	Clindamycin	--	S
Amoxicillin/ clavulanic acid	--	S	Linezolid	2	S
Oxacillin	≤ 0.25	S	Teicoplanin	≤ 0.5	S
Cephalotin	--	S	Vancomycin	≤ 0.5	S
Ceftriaxone	--	S	Doxycycline	--	S
Meropenem	--	S	Tetracycline	≤ 1	S
Gentamicin	≤ 0.5	S	Fosfomycin	≤ 8	S
Kanamycin	≤ 4	S	Fusidic acid	≤ 0.5	S
Tobramycin	≤ 1	S	Mupirocin	≤ 2	S
Ciprofloxacin	≤ 0.5	S	Chloramphenicol	8	S
Levofloxacin	--	S	Rifampicin	≤ 0.03	S
Resistance inductile to clindamycin	--	Negative	Trimethoprim/ Sulfamethoxazole	≤ 10	S

Table S10 Antimicrobial susceptibility profile of methicillin-resistant *Staphylococcus aureus* tested in Fig. 6 c, main article (test duration 10.25h)

**Antibiotic**	**MIC* µg·mL^{-1}**	**Interpretation***	**Antibiotic**	**MIC* µg·mL^{-1}**	**Interpretation***
Test Cefoxitine Screen	--	Positive	Erythromycin	1	S
Benzylpenicillin	≥ 0.5	R	Clindamycin	0.25	S
Amoxicillin/ clavulanic acid	--	R	Linezolid	2	S
Oxacillin	≥ 4	R	Teicoplanin	≤ 0.5	S
Cephalotin	--	R	Vancomycin	≤ 0.5	S
Ceftriaxone	--	R	Doxycycline	--	S
Meropenem	--	R	Tetracycline	≤ 1	S
Gentamicin	≤ 0.5	S	Fosfomycin	≤ 8	S
Kanamycin	≤ 4	S	Fusidic acid	≤ 0.5	S
Tobramycin	≤ 1	S	Mupirocin	≤ 2	S
Ciprofloxacin	≥ 8	R	Chloramphenicol	8	S
Levofloxacin	--	R	Rifampicin	≤ 0.03	S
Resistance inductile to clindamycin	--	Negative	Trimethoprim/ Sulfamethoxazole	≤ 10	S

Table S11 Antimicrobial susceptibility profile of methicillin-resistant *Staphylococcus aureus* tested in Part S15-a, ESI† (test duration 10.25h)

**Antibiotic**	**MIC* μg·mL^{-1}**	**Interpretation***	**Antibiotic**	**MIC* μg·mL^{-1}**	**Interpretation***
Test Cefoxitine Screen	--	Positive	Erythromycin	1	S
Benzylpenicillin	≥ 0.5	R	Clindamycin	0.25	S
Amoxicillin/ clavulanic acid	--	R	Linezolid	2	S
Oxacillin	≥ 4	R	Teicoplanin	1	S
Cephalotin	--	R	Vancomycin	1	S
Ceftriaxone	--	R	Doxycycline	--	S
Meropenem	--	R	Tetracycline	≤ 1	S
Gentamicin	≤ 0.5	S	Fosfomycin	≤ 8	S
Kanamycin	8	R	Fusidic acid	≤ 0.5	S
Tobramycin	≥ 8	R	Mupirocin	≤ 2	S
Ciprofloxacin	--	I	Chloramphenicol	8	S
Levofloxacin	--	I	Rifampicin	≤ 0.03	S
Resistance inductile to clindamycin	--	Negative	Trimethoprim/ Sulfamethoxazole	≤ 10	S

Table S12 Antimicrobial susceptibility profile of methicillin-resistant *Staphylococcus aureus* tested in Part S15-b, ESI† (test duration 10.25h)

**Antibiotic**	**MIC* μg·mL^{-1}**	**Interpretation***	**Antibiotic**	**MIC* μg·mL^{-1}**	**Interpretation***
Test Cefoxitine Screen	--	Positive	Erythromycin	≥ 8	R
Benzylpenicillin	≥ 0.5	R	Clindamycin	0.25	S
Amoxicillin/ clavulanic acid	--	R	Linezolid	2	S
Oxacillin	≥ 4	R	Teicoplanin	≤ 0.5	S
Cephalotin	--	R	Vancomycin	≤ 0.5	S
Ceftriaxone	--	R	Doxycycline	--	S
Meropenem	--	R	Tetracycline	≤ 1	S
Gentamicin	≤ 0.5	S	Fosfomycin	≤ 8	S
Kanamycin	≤ 4	S	Fusidic acid	≤ 0.5	S
Tobramycin	≤ 1	S	Mupirocin	≤ 2	S
Ciprofloxacin	≥ 8	R	Chloramphenicol	8	S
Levofloxacin	--	R	Rifampicin	≤ 0.03	S
Resistance inductile to clindamycin	--	Negative	Trimethoprim/ Sulfamethoxazole	≤ 10	S

***MIC**: minimum inhibitory concentration, the lowest concentration of an antimicrobial drug that will inhibit the visible growth of a microorganism after overnight incubation

***Interpretation**: R=Resistant S=Susceptible I=Intermediate

S17. Detection of antimicrobial-resistance associated proteins from two more methicillin-resistant *Staphylococcus aureus* strains

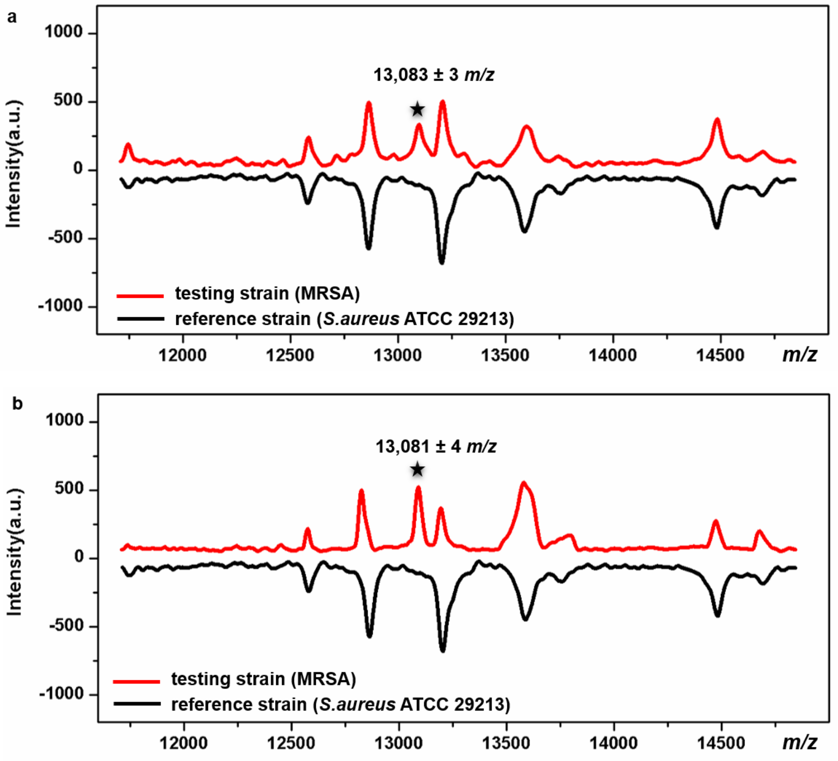

Figure S17 Detection of antimicrobial-resistance associated proteins from two more methicillin-resistant *Staphylococcus aureus* (MRSA) strains by comparing their MS fingerprint patterns with reference strain *Staphylococcus aureus (S. aureus)* ATCC 29213.

In the main article, a peak at 13,080±2 *m/z* was exclusively detected from a MRSA strain (Fig.6 c). This peak could come from the characteristic fragment of PBP 2a protein, which confers the methicillin-resistance. To further confirm this assumption, two more MRSA strains (provided by Sion Hospital, Switzerland) were tested. As shown in Figure S17, the peak around 13 kDa was also successfully detected from the two MRSA strains, at 13,083±3 and 13,081±4 *m/z*, respectively. The detailed antimicrobial susceptibility testing profiles of the two MRSA strains are listed in Table S11-12, Part S16 in this supporting document.

References

1. L. Qiao, C. Roussel, J. J. Wan, P. Y. Yang, H. H. Girault and B. H. Liu, *J Proteome Res*, 2007, **6**, 4763-4769.
2. J. Sambrook and D. W. Russell, *CSH protocols*, 2006, **2006**.
3. V. Ryzhov and C. Fenselau, *Anal Chem*, 2001, **73**, 746-750.
4. P. A. Demirev, Y. P. Ho, V. Ryzhov and C. Fenselau, *Anal Chem*, 1999, **71**, 2732-2738.
5. Y. D. Zhu, L. Qiao, M. Prudent, A. Bondarenko, N. Gasilova, S. B. Moller, N. Lion, H. Pick, T. Q. Gong, Z. X. Chen, P. Y. Yang, L. T. Loveyf and H. H. Girault, *Chem Sci*, 2016, **7**, 2987-2995.
6. R. Nakano, M. Hara, H. Ishiguro, Y. Y. Yao, T. Ochiai, K. Nakata, T. Murakami, J. Kajioka, K. Sunada, K. Hashimoto, A. Fujishima and Y. Kubota, *Catalysts*, 2013, **3**, 310-323.
